# Acknowledgement to Reviewers of *Sensors* in 2017

**DOI:** 10.3390/s18010275

**Published:** 2018-01-18

**Authors:** Sensors Editorial Office

**Affiliations:** MDPI AG, St. Alban-Anlage 66, 4052 Basel, Switzerland

Peer review is an essential part in the publication process, ensuring that *Sensors* maintains high quality standards for its published papers. In 2017, a total of 2965 papers were published in the journal. Thanks to the cooperation of our reviewers, the median time to first decision was 24 days and the median time to publication was 53 days. Among the reviewers who reviewed for *Sensors* in 2017, the following five have been selected to receive the “*Sensors* 2017 Outstanding Reviewer Award” for the timeliness and quality of their review reports in 2017:

Ciuonzo, Domenico from University of Naples, “Federico II”, Italy

Gravina, Raffaele from University of Calabria, Italy

Marques, Carlos A. F. from Aston University, UK

Mortazavi, Bobak from Texas A&M University, USA

Shojafar, Mohammad from University Sapienza of Rome, Italy

The editors also express their sincere gratitude to all of the following, who shared their time and expert opinion by reviewing for *Sensors* in 2017:
Aa, GeruoLiu, ZhiyuanAanstoos, JamesLiu-Jimenez, JudithAazam, MohamamdLlobet, EduardAbadi, Mojtaba MansourLloret Mauri, JaimeAbbamondi, Gennaro RobertoLobo-Castañón, María JesúsAbbas, SherifLocatelli, ClinioAbbasi, Qammer Lofland, SamAbd-Alhameed, RaedLöfstrand, MagnusAbdelkefi, AbdessattarLogean, EricAbdel-Motaleb, Ibrahim M.Logozzo, SilviaAbdel-Rahman, EihabLogsdon, Sally D.Abderrahmane, BenchiroufLohan, Elena SimonaAbdolee, RezaLohr, DieterAbdollahi, AmirLojou, ElisabethAbdul-Aziz, AliLombard, BrunoAbdulhalim, IbrahimLombardi, John R.Abdullah, Badr M.Long, Dan S.Abidian, MohammadLong, XiAbidin, AysajanLongo, DomenicoAbo-Serie, EssamLongstaff, Andrew PeterAbot, JandroLonini, LucaAbousleiman, RamiLopes, NunoAbramovich, HaimLópez Antón, RicardoAbu Bakr, BilalLópez De Lacalle, L.NorbertoAbuabed, Alaeddin S. A.Lopez Riquelme, Juan AntonioAbuzneid, Abdel-ShakourLopez Rodriguez, PatriciaAccettura, NicolaLópez Ruiz, NuriaAcharya, U RajendraLópez, Miguel VegaAchtert, PeggyLópez, Pedro PerisAckerley, RochelleLopez-de-Ipina, KarmeleAckermann, HannoLopez-Franco, CarlosAcosta, Antonio JoséLópez-Granado, OtonielAcuna, GuillermoLópez-Higuera, José MiguelAdali, TulayLópez-Lorente, Ángela InmaculadaAdam, VojtechLopez-Molina, CarlosAdams, JacobLopez-Otero, PaulaAdams, Matthew D.Lopez-Pellicer, Francisco J.Adebisi, BamideleLopomo, Nicola FrancescoAdhikari, Bal RamLorenz, EckehardAdivar, MuratLorenzo Abad, María EncarnaciónAdjrad, MounirLőrinc, MártonAgapiou, AthosLorite, Gabriela SimoneAgarwal, ShauryaLorussi, FedericoAgarwala, ShwetaLosada Gutiérrez, CristinaAghaie, Kiarash ZamaniLosada-Perez, PatriciaAgili, Sedig S.Loscri, ValeriaAglieri Rinella, Gianluca Loseto, GiuseppeAgostini, MatteoLosson, OlivierAgostini, ValentinaLotfi, AhmadAgranov, GennadiyLou, XinhuiAgrawalla, Bikram KeshariLouis, JulienAgu, EmmanuelLovas, TamásAguado García, DanielLovisolo, LisandroAgudo, AntonioLowe, KelseyAguilar, Sergio RíosLowe, ShehanAguilera-Servin, Juan LuisLowry, StephanieAguirre-Salado, Carlos A.Lozano Rogado, JesusAhlfors, SeppoLu, BinbinAhlström, ChristerLu, Ching-TaAhma, LuanLu, FengAhmadi, AminLu, GeyuAhmadianyazdi, AlirezaLu, Guo-WeiAhmed, FaridLu, Hai-HanAhmed, RiazLu, JianAhmed, Syed HassanLu, JiangAhn, Choon KiLu, MengAhokangas, PetriLu, Michael S.-CAhrens, JensLu, NaAhsant, BabakLu, PingAizpurua, Jose IgnacioLu, RongxingAkanyeti, OtarLu, YiAkbarzadeh, HosseinLu, YipengAkbaş, Mustafa İlhanLu, YufengAkcakaya, MuratLucas Martínez, NéstorAkhtar, ZahidLucibello, AndreaAkitsu, TetsuyaLucido, MarioAkos, DennisLucivero, Vito GiovanniAksanli, BarisŁuczak, SergiuszAlam, MuhammadLudeno, GiovanniAlam, Muhammad MahtabLudwig, FrankAlamdari, Mehrisadat MakkiLughofer, EdwinAlamgir Hossain, MohammedLui, AntonyAlan, TuncayLuini, LorenzoAlander, JarmoLukač, NikoAlapan, YunusŁukasik, SzymonAlavi, AmirLumentut, MikailAlbano, MatteoLund, AnjaAlbano, MicheleLuo, ChunboAlbarracín, RicardoLuo, Jia-NingAlbella, PabloLuo, JunhaiAlbert, Mark VLuo, QingliAlberto, GrecoLuo, ShanAlberto, NeliaLuo, SidaAlberto, Perea MorenoLuo, Tony T.Albiol-Pérez, SergioLuo, XichunAlbizu, IgorLuo, YanhuaAlbo, JonathanLuo, YishanAlbukhanajer, WissamLuo, YongAlbuquerque, Daniel FilipeLuo, YuAlcarria, RamonLuo, ZhiyuanAlcock, LisaLupan, OlegAleixandre Herrero, ManuelLupascu, DoruAlejo, DavidLupi, CyrilAlemohammad, HamedLusk, AnneAleotti, JacopoLuttge, ReginaAletta, FrancescoLuzi, GuidoAlexakis, DimitriosLv, TiejunAlexandridis, ThomasLvova, LarisaAlexandropoulos, George C.Lyakhov, AndreyAlfa, Attahiru S.Lymberopoulos, Dimitrios K.Alfano, Brigida Lyutakov, OleksiyAlfinito, EleonoraM. Garcia, NunoAl-Fuqaha, AlaMA, Andy J. Alguacil De La Blanca, GerardoMa, Andy JinhuaAli-Löytty, SimoMa, BingpengAlimenti, FedericoMa, ChangzhengAlin, MoldoveanuMa, Der-MingAlismail, HatemMa, Guo-mingAljaroudi, AliredaMa, JiajuAllasia, PaoloMa, JianguoAlluri, Nagamalleswara RaoMa, JiayiAllwood, GaryMa, JinwenAlmasri, MahmoudMa, LinglingAlmeida, AitorMa, MengChaoAlmeida, JoseMa, Xiaogang MarshalAlmeida, Luis PedroMa, XiaoleiAl-Mogren, Ahmad S.Ma, Yu TakAlmond, Darryl P.Ma, Yuan-RonAloi, GianlucaMa, YuqiangAlonso Arroyo, AlbertoMa, ZhouAlonso, Joan FMaamary, RabihAlonso-Ramos, CarlosMaccaferri, NicolòAlonzo, MichaelMaccari, LeonardoAlsadik, BasharMacDonald, EricAlshurafa, NabilMacDonald, LindsayAlsina-Pages, Rosa MaMach, PavelAl-Tahir, RaidMachado, AlencarAltet, JosepMachado, JoséAltini, MarcoMachaj, JurajAl-Turjman, FadiMacher, WolfgangÁlvarez Gil, María JoséMaciejewska, DorotaÁlvarez, J. D.Mack, BenjaminAlvarez, Juan AntonioMackay, RuthÁlvarez, MarMackenzie, JockAlvarez, RafaelMacnae, James C.Alves, JoaquimMaddahi, YaserAlves, TiagoMadeiro, FranciscoAmal, HaithamMadhu, SheriAmano, YoshiharuMaestre, RafaelAmaro, José PedroMafra, SamuelAmatya, SurajMaga, DušanAmbros, JiříMagalhães, Fabrício Anício DeAmbrož, MihaMagdziak, MarekAmezcua Correa, RodrigoMagjarević, RatkoAmezquita-Sanchez, Juan PabloMagna, GabrieleAmigo, Jose ManuelMagney, TroyAmirat, YassineMagno, MicheleAmiri, Amir MohammadMagnusson, MartinAmiruzzaman, MdMahadeva, Suresha K.Amjadian, MohsenMahajan, RuhiAmma, ChristophMahalle, Parikshit N.Ammari, HabibMahapatra, ChinmayaAmmer, KurtMahbub, Khandaker RayhanAmor, MercedesMahendran, V.Amory-Mazaudier, ChristineMahlein, Ann-KathrinAmpatzidis, DimitriosMahmud, Md. ShaadAmpeliotis, DimitrisMahshid, Sahar SadatAmundarain, AiertMainetti, LucaAmza, CatalinMaiolino, PerlaAn, NanMaiolo, LucaAn, QiMaiorana, EmanueleAn, Yun-KyuMaire, FredericAnagnostopoulos, MariosMaiwald, ChristianAnagnostou, Dimitris E.Majkowska-Skrobek, GrażynaAnanthakrishnan, Soundaram JeevarathinamMajzner, JiriAnantrasirichai, NantheeraMak, Peng UnAnastasaki, EiriniMakarona, E.Anastassiu, HristosMakhoul, AbdallahAnastassopoulos, VassilisMakhoul, EduardoAnbarjafari, GholamrezaMaksoud, TalalAnderl, ReinerMalajner, MarkoAnders, JensMalakar, NabinAndersen, Jens ChristianMalara, PietroAnderson, Brian D.O.Maldague, XavierAndersson, KarlMalek, ŽigaAndia, ManuelMalekian, RAndo, BrunoMalik, BilalAndré, PauloMalinowski, PawelAndreadis, AlessandroMalinski, TadeuszAndreescu, Gheorghe-DanielMalinverni, Eva SavinaAndreescu, SilvanaMalkin, RobAndreoni, GiuseppeMalleson, CharlesAndreotti Lage, FernandoMallios, AngelosAndreotti, FernandoMalone, Brendan P.Andreou, ChrysafisMalvezzi, MonicaAndrews, Aaron MaxwellMalviya, Kirtiman DeoAndrieux, FabriceMalykin, Grigorii B.Aneece, Itiya P.Malyuskin, OleksandrAnfossi, LauraMalzahn, JornAngani, Chandra SekharMammen, Jennifer R.Angelidis, Pantelis A.Manakhov, AntonAnggraini, Sri AyuMancini, AdrianoAnghel, AndreiMandal, BappadityaAnghelache, GabrielMandal, KrishnaAngiolini, FedericoMandal, SanghamitraAngotzi, Gian NicolaMandow, AnthonyAnia-Castanon, Juan DiegoManduchi, RobertoAnishchenko, LesyaManfred, KatherineAnnamdas, VenuMangan, MichaelAnnamdas, Venu GMMangia, MauroAnnapureddy, VenkateswarluMangili, FrancescaAnnie, ChausséManhartsgruber, BernhardAnsaloni, GiovanniMani, VeerappanAntanasijević, DavorMani, VigneshwaranAnthimopoulos, MariosManich, S.Antoine, RodolpheManickam, PandiarajAnton, DavidManikas, AthanassiosAntonacci, FabioManjarrés, DianaAntonelli, PaoloMankin, Richard W.Antonini, AlessandroMännel, BenjaminAntonini, AlessioMannini, AndreaAntonino-Daviu, Jose AlfonsoManogharan, Guha PrasannaAntonopoulos, AngelosManojlović, Lazo M.Antonopoulos, ChristosManoylov, Kalina M.Antreich, FelixMansourian, AliAntunes, MichelMante, Pierre-AdrienAnvar, Amir M.Mantiuk, RadoslawAnzai, Jun-ichiMantovani, MatteoAo, ZhiminManuri, FedericoAoyagi, TakahiroManzano, FranciscoAparicio, JoaquínManzoni, StefanoAparicio-Pardo, RamonMao, AihuaArai, YasuoMao, HanbinArami, ArashMao, LeiArandjelović, OgnjenMao, YuxingAranha, JoseMaraloiu, Valentin-AdrianArani, ArvinMaran, ViniciusAraromi, OluwaseunMarapareddy, RamakalavathiArash, BehrouzMarasso, SimoneArastounia, MostafaMarathe, AmarAraujo Dos Santos, José ViriatoMarathe, ThyagarajaAraujo, AlvaroMarcelli, RomoloAraujo, FilipeMarcello Ruiz, JavierAraujo, GustavoMarcelloni, FrancescoAraújo, Rui EstevesMarchamalo Sacristan, MiguelArazuri, SilviaMarchetti, MarioArbabi, VahidMarcolin, FedericaArena, FrancescaMarcolino, Leandro SorianoAretakis, NikolaosMarczewski, JacekArgenti, FabrizioMardanbegi, DiakoArgia, KatsuhikoMaree, RaphaelAribisala, BenjaminMarfievici, RamonaAricò, PietroMariani, StefanoArjunan, Sridhar PoosapadiMarichy, CatherineArmada, ManuelMarin, AndreaArmendáriz, Marco Antonio MorenoMarinello, FrancescoArmijo, FranciscoMarinić-Kragić, IvoArnay, RafaelMarino, ArmandoArnold, F. J.Marino, StefanoArribas, JavierMarinoni, MauroArriola, AitorMarkakis, Evangelos K.Arroyo, NetzahualcóyotlMarkevičius, VytautasArrue, Begoña C.Markl, DanielArtale, ValeriaMarkos, ChristosArtemev, AndreiMarks, Haley L.Artese, GiuseppeMarks, MichalArtola, LaurentMaroufi, MohammadArvanitis, KonstantinosMarozas, VaidotasArya, SunilMarquardt, DrewAryan, PouriaMarques Da Silva, JoséArzuaga, EmmanuelMarques, Carlos A. F.Asadnia, MohsenMarques, Manuel Joaquim BastosAscroft, HelenMarrazza, GiovannaAshjaei, MohammadMarrero, DomingoAshley, JonMarrone, StefanoAskoxylakis, Ioannis G.Mars, KamelAslani, FarhadMarschner, SteveAslanides, Ioannis M.Marsh, Eric R.Asorey-Cacheda, RafaelMarta, Ferreiro-GonzálezAsprou, MarkosMartalò, MarcoAssimonis, Stylianos D.Martel, SylvainAssunção, PedroMartín Gómez, DavidAstrain, José JavierMartin, RussellAtalay, OzgurMartin, Tara J.Atchison, Justin A.Martinek, RadekAthanasiou, LamprosMartinez De Dios, José RamiroAthanasopoulos, NikolaosMartínez De Paz, Pedro JoséAtia, MohamedMartinez Espinosa, Juan CarlosAtkinson, Gary M.Martinez Hardigree, JosueAttarzadeh-Niaki, Seyed-HoseinMartinez Perez, MariaAttoh-Okine, NiiMartínez Torres, JavierAttux, RomisMartínez, AntoniAtzori, ManfredoMartinez, Ramses V.Au, ManMartinez-Hurtado, J.L.Aubin, PatrickMartinez-Izquierdo, EstibalizAubry, AugustoMartínez-Sánchez, JoaquínAugustyniak, PiotrMartinez-Triguero, JoaquinAung, Myat-Thu-LinnMartini, AlbertoAuvinet, EdouardMartini, DougAu-Yeung, Ho YuMartin-Lopez, SoniaAval, Yashar M.Martino, LucaAvanzi, FrancescoMartino, SalvatoreAvila, KerstinMartinovic, IvanAviles-Rivero, Angelica I.Martins, Felipe NascimentoAvrutsky, IvanMartins, João P. A.Aw, KeanMartins, RodrigoAxenie, CristianMartins, Wallace AlvesAYE, Su YeeMartín-Sacristán, DavidAyerden, N. PelinMartinsen, Ørjan G.Ayers, Paul D.Martins-Filho, Luiz S.Azam, Saeed EftekharMartín-Yerga, DanielAzar, Ahmad TaherMartowicz, AdamAziz, Syed MahfuzulMartynov, DenisAzpilicueta, LeyreMarzano, AdelaideBabadi, BehtashMarzbanrad, FaezehBabaie, MasoudMarzocca, CristoforoBabanajad, SaeedMasayuki, AnyojiBaber, ChrisMashima, DaisukeBabiarz, A.Masiero, AndreaBaboli, MehranMasini, Barbara MaviBabu, R. JayachandraMasłowski, PiotrBacciu, DavideMason, AlexBackhouse, ChrisMasotti, DiegoBadawy, MoawadMassa, AndreaBadea Doni, MihaelaMassad-Ivanir, NaamaBadescu, Alina-MihaelaMassaouti, MariaBadger, MereteMassaroni, CarloBadihi, HamedMastmeyer, AndreBadilescu, SimonaMastriani, MarioBae, EuiwonMatagne, ErnestBae, Hyung DaeMatatagui, DanielBae, Ihn-hanMatei, BasarabBae, JinhoMatekovits, LadislauBae, YoungchulMateo-Lázaro, JesúsBaek, Chang-KiMateos, CristianBaek, JoonsangMateos, IgnacioBafekrpour, EhsanMaterazzi, Annibale LuigiBaghdadi, NicolasMatharu, ZimpleBaghdasaryan, TigranMathew, BobbyBaghernejad, MasoudMathews, SunishBahoura, MohammedMathwig, KlausBai, LuMatko, VojkoBai, XueruMatsui, TakemiBai, YongMatsumoto, SatoshiBailey, Sean C.Matsumura, KentaBailly, Jean-StéphaneMatsushita, KojiroBailón, RaquelMatsuura, MotoharuBaiocchi, ValerioMatsuura, YujiBaker, TharMattarelli, MaurizioBakillah, MohamedMaudes, JesusBakuła, KrzysztofMauricio, JoanBakuła, MieczysławMaurits, Natasha M.Bala, CameliaMavridis, NikolaosBalaji, BharathanMavromatidis, LazarosBalal, EsmaeilMay, StefanBalali, VahidMaybank, SteveBalasingam, BalakumarMayer, ChristianBalduzzi, MarcoMayer, ChristopherBalid, WalidMayorov, AlexanderBallesta-Claver, JulioMazeika, DaliusBally, MartaMazomenos, EvangelosBalster, Eric J.Mazuelas, SantiagoBanan Sadeghian, RaminMazurek, PrzemysławBanas, Neil S.Mazza, ClaudiaBandodkar, Amay JairajMazzaro, GregoryBanerjee, PratikMazzei, DanieleBanerjee, TanviMazzetto, FabrizioBanitsas, KonstantinosMazzoni, AugustoBannan, BrendaMbarek, NaderBao, JingjingMcCaig, Heather Bao, RongrongMcCall, CadeBao, YiMccarty, Gregory S.Baqersad, JavadMcCaul, MargaretBarabutis, NektariosMcCool, ChristopherBaran, RemigiuszMcDaniel, TroyBaran, UtkuMcDonald, AnthonyBarandiaran, IñigoMcDonald, TimothyBaranowska-Korczyc, AnnaMcFadyen, AaronBarbarin, YohanMcFall, KevinBarbato, MarcoMcFarlane, NicoleBarbeau, MichelMcGilvray, KirkBarber, RamónMcGinnis, Ryan S.Barberis, AntonioMcgookin, EuanBarbiellini, BernardoMcGuire, MichaelBarbosa, Ramiro S.McKeague, MaureenBarbosa, Roberto SpinolaMcKeating, Kristy S.Barclay, DavidMcKeever, SusanBardhan, NeelkanthMckelvey, KimBarille, RegisMckenzie, IainBarmpounakis, Emmanouil N.McLaughlin, StephenBarnard Feeney, Allison McLeod, EuanBarnard, Ross T.Meade, Aidan D.Barnett, A. M.Mech, KrzysztofBarniol, NuriaMechbal, NazihBarone, Pier MatteoMedeiros, HenryBarr, Richard G.Medina, Manuel AdamBarralon, PierreMedina-Plaza, CristinaBarreca, SalvatoreMeditskos, GeorgiosBarrell, JeffreyMedjroubi, WidedBarrera, FernandoMedley, AnnBarrett, BrynleMedola, FaustoBarrett-Gonzalez, RonaldMedrano, CarlosBarsan, Madalina M.Medrano, NicolásBarsocchi, PaoloMedved, VladimirBartoletti, StefaniaMedvidović-Kosanović, MartinaBartonova, AlenaMeek, Sanford G.Barzilov, AlexanderMegias, DavidBaś, BogusławMegret, PatriceBas, JoanMehmood, RashidBasco, Leonardo K.Mehnen, JornBasiri, AnahidMei, LeiBassford, MarieMeisen, TobiasBastakoti, Bishnu PrasadMekikis, Prodromos-VasileiosBastawrous, Hany AyadMeldrum, Tyler K.Bastian, OlafMeli, EnricoBastide, RémiMelillo, PaoloBastos, Maria LuísaMelnikau, DzmitryBastos-Filho, TeodianoMelnykowycz, MarkBasu, AnirbanMelo, JoséBáťková, KamilaMeloni, AlessioBattaglia, AlessandroMelo-Pinto, PedroBattistelli, CeciliaMelvin, William L.Bauer, RomanMena, EduardoBaulin, Vladimir A.Mendes, LuísBaumbach, Jörg IngoMéndez Fuentes, ValerianoBayford, Richard H.Méndez, Alexis J.Bayse, Craig A.Mendez, DiegoBazeille, StephaneMendizabal, JaizkiBazzi, AlessandroMenendez, HectorBazzu, GianfrancoMeneses, FilipeBeauchamp, JonathanMeng, GuangBęben, AndrzejMeng, GuowenBechelany, MikhaelMeng, LeiBechhoefer, EricMeng, LinBecker, ClemensMeng, ZhaozongBecker, Donald F.Meng, ZhiguoBecker, MarceloMengistie, Desalegn AlemuBecker, MartinMeo, MariannaBeckerle, PhilippMeola, CarosenaBedogni, LucaMerchant, Gina C.Begušić, DinkoMerciol, FrançoisBeheim, Glenn M.Mercorelli, PaoloBehmann, JanMerino, LuisBel, AlbertMerino, SilviaBelagiannis, VasileiosMerlen, AlainBelal, MohammadMerlo, SabinaBelay, Gebirie YizengawMesa, Juan A.Belen, DiezmaMesas-Carrascosa, Francisco JavierBelgiu, MarianaMeschino, SimoneBelisario-Briceño, AndresMescia, LucianoBellekens, XavierMeseguer, RocBellido, Francisco J.Meshgi, KouroshBelloch, Jose AntonioMesquita, EsequieBellvert, JoaquimMessina, FabrizioBeltrame, GiovanniMessina, MarcoBeltran, ChrisMessori, LuigiBeltrán, Vanesa SanzMestha, Lalit KBelyaev, EvgenyMestre, DanielBen Abdallah, AbderazekMetsis, VangelisBen Simon, EtiMetzger, Robert M.Ben, YueyangMeulenberg, ElineBenardos, PanoriosMeyer, AlexandreBenato, AlbertoMeyners, DirkBenatti, SimoneMeza, IvanBenchabane, SarahMiah, Abdul HalimBendavid, YgalMiah, KhalidBendek, EduardoMiao, GuoxingBender, FlorianMichail, ChristosBender, PaulMichal, CarlBenedetti, IvanoMichalski, AndrzejBenedicenti, LuigiMichard, FredericBenedict, KarlMichel, AnnaBeneš, PetrMichelena, MarinaBenetti, MassimilianoMicheli, DavideBenghazi, KawtarMicheli, LauraBenhabiles, HalimMichelson, DavidBeni, ValerioMichimura, YutaBenjamin, OfirMicic, MiodragBen-Moshe, BoazMieloszyk, MagdalenaBennetts, Victor HernandezMien, VanBenoussaad, MouradMiguel, Gonzalo DeBerardi, LuigiMiguel, SilviaBerbecaru, DianaMihajlović, VojkanBerényi, AntalMihara, TsuyoshiBerge, Therese WithMihaylova, LyudmilaBergeles, ChristosMihovska, Albena D.Bergeot, NicolasMikhaylov, KonstantinBergmann, Neil W.Miki, HirofumiBergmann, OlafMikita, TomášBergonzo, PhilippeMiklavcic, StanBeriáin, AndoniMikš, AntonínBermudez Edo, MariaMilazzo, CristinaBermudez-Cameo, JesusMiled, AmineBernabucci, IvanMileti, GaetanoBernard, YvesMilillo, PietroBernardi, GiulioMilitino, Ana FernándezBernardino, JorgeMiller, BorisBernardos, Ana M.Milosevic, BojanBernardos, AndreaMilovanovic, BojanBernasconi, GiancarloMin, ChangjunBernassau, AnneMin, GeyongBerndt, PhillipMinami, Tsuyoshi Berneschi, SimoneMinamikawa, TakeoBernier, MartinMinamiki, TsukuruBernini, RomeoMinardo, AldoBertacchini, AlessandroMinas, GracaBertocci, FrancescoMinervini, MassimoBertoncello, PaoloMinghini, MarcoBesada, EvaMiniaci, MarcoBétaille, DavidMinonzio, Jean-GabrielBetancourt, DiegoMirabella, SalvoBettinger, PeteMirabello, VincenzoBeutel, SaschaMiraglia, FrancescaBevacqua, MartinaMiragliotta, GiovanniBeverini, NicolòMiranda, CarlosBevilacqua, MartaMiranda-Castro, RebecaBeyeler, MichaelMirbozorgi, AbdollahBeyerer, JürgenMiridonov, SergueiBeyerle, GeorgMiron, SebastianBezryadina, AnnaMiroshnichenko, AndreyBhagat, YusufMirsky, VladimirBhalla, NikhilMirza, FarhaanBhandari, SubodhMirzaee Kakhki, Iman Bhatia, SajalMirzaei, GolrokhBhattacharjee, TapomayukhMirzavand, RashidBhattarai, NishanMishne, DavidBhotto, Md. Zulfiquar AliMishra, AmitabhBhuiyan, Mohammad Zahidul H.Mishra, Kumar VijayBi, KeMishra, Rupesh K.Biagetti, GiorgioMisiakos, KonstantinosBialkowski, KonstantyMišić, Vojislav B.Bian, JiangMiskowicz, MarekBianchi, Anna MariaMitchell, JohnBibbo, DanieleMitchell, SteveBibo, AminMitobe, KazuhisaBicen, A. OzanMitra, SunandaBień, AndrzejMiura, HiroyukiBieniek, TomaszMiwa, TomioBijelić, GoranMiyamori, YasunoriBikbova, GuzelMiyazaki, HiroyukiBikov, AndrásMizsei, JánosBilleci, LuciaMo, JinhanBilotta, EleonoraMoafipoor, ShahramBilotta, GiulianaMobashsher, Ahmed ToahaBinietoglou, IoannisMobed, ParhamBirch, BrianMobed-Miremadi, MaryamBircher, SimoneMocanu, Irina GeorgianaBird, JonMochaourab, RamiBirlan, MirelMock, AdamBischof, Hans-PeterMoczulski, WojciechBischofs-Pfeifer, IlkaModotto, DanieleBisegna, FabioMogan, GheorgheBisio, IgorMoh, SangmanBitter, MartinMohammadi, FatemehBizzi, SimoneMohammed Salem, Mohammed Abdel-MegeedBlaabjerg, FredeMohammed, Beadaa JasemBlackman, ChristopherMohd-Yasin, FaisalBlais, AntoineMöhring, Hans-ChristianBlake, CatherineMoissenet, FlorentBlanc, SaraMokhtari, GhassemBlanch, JuanMokhtari, SoroushBlanco, Jose LuisMolazemhosseini, AlirezaBlankenship, ClayMölders, NicoleBlasco, JorgeMoll, JochenBlasi, JuanMolleda, JulioBłaszczak-Bąk, WioletaMomeni, KasraBleichner, MartinMomm, HenriqueBleser, GabrieleMonasse, PascalBlin, StéphaneMondal, KunalBlock, StephanMondal, Tapas K.Blomqvist, EvaMondin, MarinaBlondeau-Patissier, VirginieMongus, DomenBludov, YuMonico, João Francisco GaleraBlue, RobertMonkawa, AkiraBlumenstein, JiriMönks, UweBlumrosen, GaddiMonrroy, JavierBlustein, DanMonserrat, OriolBoada, Beatriz L.Monsivais-Huertero, AlejandroBoada, Maria Jesus LopezMontanini, LauraBoano, Carlo AlbertoMontavont, JulienBobis, OtiliaMontecucco, AndreaBobowski, JakeMonteiro Santos, Fernando A.Bobulski, JanuszMonteiro, JoãoBochenkov, Vladimir E.Montero, R. SánchezBochud, NicolasMontez, CarlosBodini, IleanaMonti Guarnieri, AndreaBodnar, Jean LucMontoliu Colas, RaulBoelt, BirteMontuori, AntonioBoër, MichelMonzón, Mario D.Bogdan, IleanaMook, GerhardBogdan, PaulMoon, Joo HyunBohlman, StephanieMoon, SangJunBoisseau, SebastienMoore, PhilipBojkovski, JovanMoothanchery, MoheshBoland, ConorMora, AntonioBoletsis, CostasMora, HiginioBolkas, DimitriosMorabito, Francesco CarloBölöni, LadislauMorais Dos Santos, RaulBölöni, LotziMorales, Elizabeth RendonBolotov, LeonidMorales, Francisco Javier OrdonezBolshov, Mikhail A.Morales-Narváez, EdenBolton, JeremyMoran, KieranBonanno, AlbertoMoran-Mirabal, JoseBonastre Pina, Alberto MiguelMorbidi, FabioBonenberg, LukaszMoreau, JulienBonfè, MarcelloMoreira, Adriano Jorge CardosoBoniface, KarenMoreira, Felismina T.C.Bonifacio, AloisMoreira-Matias, LuisBonizzi, PietroMorell, AntoniBonnabel, SilvèreMorell, GerardoBoragno, CorradoMoreno Arboleda, Francisco JavierBoratto, LudovicoMoreno, Donato LunaBorbone, NicolaMoreno, FélixBorboni, AlbertoMoreno, IvanBorecki, MichalMoreno, MiguelBorghetti, BrettMoreno, PabloBorghi, AlessandraMoreno-Garcia, Isabel MaríaBoric-Lubecke, OlgaMoreno-Salinas, DavidBorio, DanieleMoreno-Valenzuela, JavierBorisoff, JaimieMoretti, ElisaBorkowski, AndrzejMoretti, SandroBorkowski, PiotrMorgado, LeonelBörner, AnkoMori, NobuyaBorri, SimoneMori, SaverioBorschbach, MarkusMorillo, CarlosBortolotti, CarloMorimitsu, HenriqueBortot, ElianaMorineau, ThierryBoscariol, PaoloMorioka, KazuyukiBosch Ruiz, MarcMORISAWA, YusukeBosch, StephanMoritz, Guilherme LuizBosisio, Ada VittoriaMorley, Nicola ABosse, StefanMoron, CarlosBotelho, Bruno G.Morón, M. J.Botha, Elizabeth JohannaMoroni, DavideBothos, EfthimiosMoroni, MonicaBotzheim, JánosMorosi, SimoneBoubchir, LarbiMorozov, MaximBou-Cabo, ManuelMorozs, NilsBouchard, KevinMorrey, DeniseBouckaert, JulieMorris, BrendanBoudaden, JamilaMorris, Gary A.Bouk, Safdar HussainMorris, TimBoulogeorgos, Alexandros-Apostolos AMorrison, KeithBouras, ChristosMorshed, BashirBousia, AlexandraMortazavi, BobakBousquet, Jean-FrançoisMortello, MichelangeloBoutaayamou, MohamedMorton, CraigBoutayeb, HalimMorzaria Luna, Hem NaliniBoutejdar, AhmedMoscato, VincenzoBouvet, MarcelMoschitta, AntonioBouvet, Pierre-jeanMoscone, DanilaBouwmans, ThierryMoseke, ClausBoverman, GregMoshou, DimitriosBowen, JamesMossa, MicheleBowker, B. C.Mossin, SusanneBowles, Jeffrey H.Mostarda, LeonardoBoyacı, İsmail HakkıMotai, YuichiBoyd, DarrylMotta, GianmarioBoyer, MarcMotti, Vivian GenaroBoysen, ReinhardMotto Ros, PaoloBradley, Justin M.Moura, JoseBradley, Patrick ErikMourad-Chehade, FarahBraeken, AnMourdikoudis, StefanosBraga, Guilherme S.Mourtzis, DimitrisBrandão, AlexandreMousavi Lajimi, S. AmirBrandao, PedroMousavi, PedramBrandl, MartinMousavi, S. MostafaBrás, SusanaMousavi, ZekraBratus, SergeyMoussa, AdelBräuer-Burchardt, ChristianMoussa, SherifBraun, AndreasMoussavi, Zahra KazemBrazhe, NadezdaMowla, AlirezaBrčić, DavidMrochen, MichaelBreglio, GiovanniMu, LuyeBrehar, RalucaMück, MichaelBretenaker, FabienMuehlsteff, JensBreunig, IngoMueller, MarkBrice, SorliMuharam, Farrah MelissaBridle, HelenMühlbacher-Karrer, StephanBrigham, KatharineMukherjee, AnondoBrilly, MitjaMukherjee, DibyenduBrinkworth, RussellMukherjee, Partha SarathiBrischetto, SalvatoreMukherjee, SoumyaBrito-Loeza, CarlosMuley, PranjaliBrock, StefanMulianga, BettyBrodnick, SarahMuller, AlexandruBrolly, MatthewMuller, CobusBronzino, FrancescoMüller, ThomasBrook, AnnaMuller-Landau, HeleneBrooks, CalebMüllerová, JanaBrosed, Francisco JavierMulloni, VivianaBrouzgou, ΑngelikiMumolo, EnzoBrown, StephenMunafo, AndreaBrown, Tim W. C.Munaretto Fonseca, AneliseBrown, TimothyMunishwar, Vikram P.Brownlee, NevilMuniz-Miranda, FrancescoBrownlee, SandyMuniz-Miranda, MaurizioBroza, YoavMuñoz, AsunciónBruck, Hugh A.Muñoz, EstefaniaBrückl, HubertMuñoz, JoseBruckner, FlorianMuñoz-García, Miguel A.Bruckner, GudrunMuñoz-Gea, Juan PedroBruder, StephenMuñoz-Organero, MarioBrugarolas, RitaMunschy, MarcBruggi, MatteoMünster, PetrBrumfield, Brian E.Munteanu, Mircea GhBruna, ArcangeloMunz, MichaelBruniecki, KrzysztofMura, AndreaBrunner, AndreasMuralikrishnan, BalaBruno, ElenaMurayama, RiichiBruno, Thomas J.Muroyama, MasanoriBrust, Matthias R.Murphy, CarolineBrzozowska, EwaMurphy, James M.Bucca, GiuseppeMurroni, MaurizioBuch, Anders GlentMurtra, Andreu CorominasBuchman, AttilaMuscato, GiovanniBudgett, DavidMusić, JosipBudzier, HelmutMusleh, BasamBueno-Martinez, AntonioMuslimov, EduardBuhl, Josef-ChristianMusolino, AntoninoBuhot, ArnaudMussetta, MarcoBui, Minh-PhuongMuth, John FBui, NicolaMuthukumar, SriramBukkapatnam, Satish T.S.Myers, MikeBulić, NevenMyre, JosephBull, PeterMyrick, Michael L.Bunea, Alina-CristinaNa, Wongi S.Bunge, Christian-AlexanderNaceri, AbdeldjallilBuono, AndreaNadeem, TamerBuonocore, FrancescoNaderi, M. YousofBurattini, LauraNaeem, UmairBurch, MichaelNagai, MasayaBurdzik, R.Nagaraju, ManoharBurgdorf, MartinNagayama, TomonoriBurgos, MateoNahar, Niru K.Burian, AdinaNaik, GaneshBuric, MichaelNájera López, AlbertoBurnett, AndrewNajibi, NasserBurns, BrendanNakagaki, KenBurrascano, PietroNakamura, RyoheiBurud, IngunnNakanishi, MasakiBusche, ChristophNakano, TadashiBustillo, AndresNakashima, ShotaButt, HaiderNakatani, TomoyaButun, IsmailNakatsuma, KeiBykov, AlexanderNakayama, MasashiByl, Nancy NNakhmani, ArieBystrov, AleksandrNakonieczna, AnnaCaballero-Gil, CándidoNalbach, PeterCaballero-Gil, PinoNalepa, JakubCabboi, AlessandroNallala, JayakrupakarCabral, JaderNam, InhoCabral, JorgeNam, Su ManCabrera Carrillo, Juan AntonioNama, NiteshCaccavale, FabrizioNamieśnik, JacekCaesarendra, WahyuNamiot, DmitryCagnoni, StefanoNamuduri, KameshCahalane, ConorNanayakkara, ThrishanthaCai, LeNandi, DipCai, LinNannen, VolkerCai, ZhipengNanni, LorisCai, ZhongyuNanzer, JeffreyCaimmi, MarcoNapoli, AlessandroCain, StephenNaqvi, Syed MohsenCaizzone, StefanoNarakathu, Binu BabyCakmak, OnurNaranjo, JoseCalabretta, Maria MaddalenaNaranjo-Hernández, DavidCalado, João M. F.Nardini, GiovanniCalbimonte, Jean-PaulNaruniec, JacekCaldas, PauloNascetti, AndreaCaldeira, Joao Manuel L. P.Naseer, NomanCaleb-Solly, PramindaNasimuddin, NasimuddinCaleffi, MarcelloNasir, SaeedCalera Belmonte, AlfonsoNasiriavanaki, MohammadCalero, Olga CaballeroNassar, SamehCalì, MicheleNasseri, M. AliCaliendo, CinziaNassiri, SomayehCalleja, MontserratNatale, VincenzoCallejon, M. AmparoNatsuaki, RyoCalokerinos, AntonyNaumann, StefanCalonico, DavideNavaei, MiladCalveras, AnnaNavarro Ruiz, Juan MiguelCalvo Rolle, Jose LuisNavarro, Javier LorenzoCalvo, RoqueNawar, SaidCam, HasanNawrat, AleksanderCamargo Junior, Joao BatistaNaya Villaverde, Miguel ÁngelCambi, MartinaNdieyira, JosephCampbell, MichaelNebel, Jean-christopheCampopiano, StefaniaNeculoiu, DanCampos, RicardNef, TobiasCampos, RuiNegro, FrancescoCamps, IhosvanyNegru, MihaiCampuzano Ruiz, SusanaNehorai, AryeCan, ArnaudNeil, DanielCandanedo, Luis MiguelNejman, MirekCanessa, AndreaNele, LuigiCañete, Francisco JavierNelson-Cheeseman, BrittanyCano Garcia, Jose ManuelNemova, GalinaCano, Juan LuisNeri, GiovanniCanteli, Alfonso FernándezNeri, MatteoCao, ChangyongNesterova, Irina V.Cao, GuohuaNeto, Armando AlvesCao, HungNeto, PedroCao, JialanNeubert, JeremiahCao, KaiNeuenschwander, JuergCao, SiyangNeuman, B. CliffordCao, Wen-PingNeumann, HeikoCao, XianghuiNeveln, IzaakCao, YueNewbury, NathanCao, ZongjieNewe, ThomasCao-Paz, Ana MaríaNewlands, Nathaniel K.Capineri, LorenzoNewman, Kimberly E.Caporaso, NicolaNg, AlphonsusCappelli, ClaudiaNg, AndyCapra, AlessandroNg, Ching-TaiCapria, AmerigoNg, Derrick Wing KwanCapuano, RosamariaNg, Sum HuanCapuano, VincenzoNg, Wai TungCaputo, RobertoNgo, Chun Yong AndrewCaramés, Tiago FernándezNgo, Hoan ThanhCarballar, AlejandroNgo, VienCarbonaro, NicolaNgoko, YanikCarbone, GiuseppeNgomo, SuzyCarbone, MarinaNguang, Sing KiongCardenas-Juarez, MarcoNguyen, Duong VanCardinaels, RuthNguyen, Luan T.Cardinal, Maria FernandaNguyen, Minh TuanCardinal, PatrickNguyen, Nam-TrungCarette, JérômeNguyen, Ngoc HungCarlen, Edwin T.Nguyen, Ngoc Tu (Ryan)Carles, GuillemNguyen, T. HienCarmo, JoãoNguyen, ThuyCarmona-Murillo, JavierNguyen, Trong-TheCarrasco, SergioNguyen, TuyenCarrascosa, CarlosNi, DalongCarreno-Luengo, HugoNi, Melody (Zhifang)Carrera Barroso, Álvaro Ni, YangCarro Fernandez, GermánNico, GiovanniCarroll, MarkNicoletta, Fiore PasqualeCaruso, AntonioNie, LimingCarvajal, Miguel AngelNie, MengyanCarvalho, HelderNiederleithinger, ErnstCarvalho, LidiaNiedziolka-Jönsson, JoannaCarvalho, PauloNielsen, JohnCarvalho, VítorNielsen, KristianCasagrande, DanieleNieto, AnaCasaletti, MassimilianoNiggemann, OliverCasals, AlíciaNigussie, EthiopiaCasals-Terré, JasminaNiitsu, KiichiCasanova, RaimonNijsure, YogeshCasari, PaoloNikolakopoulos, KonstantinosCasas Rius, Joan RamonNikolaou, SymeonCasciati, FabioNikolelis, Dimitros P.Caselli, FedericaNiku, SaeedCasilari Pérez, EduardoNilfouroushan, FaramarzCasimiro, AntónioNilsson Plymoth, AndersCasinovi, GiorgioNilsson, Hans-ErikCaso, GiuseppeNima, ZeidCasquel, RafaelNimmo, FrancisCastagnola, ElisaNims, Douglas K.Castañeda, CarmenNimse, Satish BalasahebCastano, Lina M.Ning, ZhiCastellazzi, GiovanniNino, PasqualeCastiglione, AnielloNinomiya, SeishiCastiglione, ArcangeloNishar, AbdulCastilla, GuillermoNishijima, YoshiakiCastillejo, PedroNishimura, HarukiCastillo Sequera, José LuisNishio, MayukoCastro Do Amaral, GustavoNishio, YoshifumiCasula, GiuliaNishiyama, MichikoCatalá, AlejandroNisisako, TakasiCatalá-Civera, Josè ManuelaNissen, IvorCatalao, JoaoNissinen, IlkkaCatalin, PetrescuNitti, MicheleCataliotti, AntonioNiu, HaiqiangCatanante, GaelleNiu, LeiCatapano, IlariaNiwano, MichioCatherwood, Philip A.Noack, BenjaminCattelani, LucaNoak, BenjaminCavalli, Rosa MariaNobach, HolgerCavallotti, PietroNocerino, EricaCavenaghi, MarcosNogales-Bueno, JulioCazorla, MiguelNoghanian, SimaCazzulani, GabrieleNorouzi, HamidCegla, Frederic Norouzpour-Shirazi, ArashkCelebi, EmreNorrdine, AbdelmoumenCelenk, MehmetNorte, RichardCelesti, AntonioNorton, MichaelCelikin, MertNossol, EdsonCellini, FilippoNotarpietro, RiccardoCellini, NicolaNovák, PetrCenek, MartinNovellino, AlessandroCennamo, NunzioNovitzky, MichaelCenten, PeterNovoa, StéfaniCepuder, PeterNóvoa, X. RamónCerda, FernandoNóvoa-Muñoz, Juan CarlosCerdà-Alabern, LlorençNowaczyk, SławomirCernak, MilosNowak, AleksanderČernÝ, MartinNowosielski, AdamCerrone, CarmineNoyel, GuillaumeCervantes, JairNtalampiras, StavrosCesano, FedericoNtotsios, EvangelosCetin, Arif EnginNugen, Sam R.Cetó, XavierNunes, FernandoCevenini, LucaNunes, Nuno JardimCeyssens, FrederikNúñez Carmona, Estefanía Cha, Young-JinNúñez Vicencio, AlfredoCha, YoungsuNuñez, NeftaliChacon, RolandoNúñez, PedroChadli, MohammedNurchis, MaddalenaChady, TomaszNyka, KrzysztofChai, RifaiNykiel, GrzegorzChai, XinyuO' Keeffe, SineadChakeri, AlirezaO’Mahony, ConorChakraborty, NilayOballe-Peinado, ÓscarChalioris, ConstantinObata, KentaChalvatzaki, GeorgiaObeid, SimonChan, Chia-TaiO'Brien, Megan K.Chan, ChungObst, MarcusChan, Eddie C. L.Occhipinti, LuigiChan, PakOchoa, Sergio F.Chan, Stanley H.Ocket, IljaChandra, Kumar P. BharaniOcłoń, PawelChandra, Shekhar S.O'Connell, EoinChandrahalim, HengkyOdabasi, HayrettinChang, Chao-TsunOdijk, DennisChang, Ching-LungO'Donnell, TerenceChang, Fuh-YuO'Driscoll, CillianChang, Herng-huaOelmann, BengtChang, HoOesterschulze, EgbertChang, Hsing-ChengOggioni, AlessandroChang, Hsueh-HsienOgihara, NaomichiChang, IpinOgino, ShujiChang, JingboOgurtsov, VladimirChang, JinhoOh, Beom-SeokChang, JunOh, HoonChang, JunghwanOh, Sang-HyunChang, Kai-ChunOh, Tae-KeunChang, Kang-MingOhira, Shin-IchiChang, Kuo-JenOhodnicki, PaulChang, Min-KuanOhtsu, YasunoriChang, Rwei-ChingOhya, HiroyoChang, Shao-HsiaOikonomou, GeorgeChang, Sheng-PoOikonomou, KonstantinosChang, Suk TaiØiseth, Ole AndreChang, TaiwooOjeda, LauroChang, Te-WeiOkamoto, ShogoChang, Wei-ChiaoOkarma, KrzysztofChang, Yao-JenOkazaki, ShinjiChang, Yau-ZenO'Keefe, StevenChang, YoungOken, Barry S.Chang, Yuan-JenOkochi, MinaChang, ZhengOkubo, MasaakiChangshan, WuOkumura, ShiroChao, TongOlaizola, Igor GarcíaChaour, IssamOlaru, AndreiCharbon, EdoardoOlasagasti, FelixChardon, GillesOldeland, JensChargé, PascalOldham, KennCharlton, PeterO'Leary, RichardCharlton, Peter ChristopherOlejnik, PawełChatterjee, MainakOletic, DinkoChatti, KiranamOlguin, LuisChaturvedi, ItiOliveira Salles, MaiaraChaturvedi, VikramOliveira, HenriqueChatzi, EleniOliveira, LuísChatzigiannakis, IoannisOliveras, Paz FernándezChau, Kwok-wingOlofinjana, AyodeleChauhan, VedangOlson, BritaChe, WeitaoOlson, Michael W.Chen, BadongOlyaee, SaeedChen, BaiOMAR, MawloudChen, BinqiangOmura, YasuhisaChen, BoO'Neil, GlenChen, Bo-HaoOnen, OnursalChen, ChenOneto, LucaChen, Cheng-HuanOng, BoonChen, ChiachungOng, Lay TeenChen, Chia-YenOniga, ErsiliaChen, Chien-FuOniga, FlorinChen, Chien-ShengOo, Aman ThanChen, Chi-HuaOommen, ThomasChen, CunjianOpdahl, AricChen, DaiqinOpromolla, RobertoChen, Denis GuangyinOrazbayev, BakhtiyarChen, Der-chinOrbach, RonChen, Gang S.Orchard, GarrickChen, HaimingOriot, Helene M.Chen, HongyangOriwoh, EdewedeChen, HowardOrlandi, AntonioChen, HuifangOrlando, FrancescaChen, Jen-JeeOrniana Ancuti, CodrutaChen, JiajunOrosz, GyörgyChen, JiaweiOrozco Barbosa, LuisChen, JuhongOrsini, AndreaChen, JunpingOrte, AngelChen, KongzheOrtega, Francisco R.Chen, Kuan-ChungOrtiz Salazar, AndresChen, Kuan-FuOrtiz, AlfredoChen, LeiOrtiz, RobertoChen, LiangOrtiz-García, MarioChen, Lien-WuOrtmanns, MauritsChen, LijiOsaba, EnekoChen, LiminOsechas, OkuaryChen, LingxinO'Shaughnessy, SeamusChen, Lung-ChienOssadtchi, AlexeiChen, MinOstrowska, KseniaChen, MingzhouOsuagwu, BethelChen, NengchengOsuch, TomaszChen, PengO'Sullivan, ThomasChen, PengyuOsuna-Enciso, ValentínChen, QiangOswald-Tranta, BeateChen, QiushuiOta, HirokiChen, Roland K.Ota, KaoruChen, RongzhangOterkus, ErkanChen, RuiminOtis, MartinChen, SanjianOtnes, RoaldChen, Shao-WenOtomański, PrzemysławChen, Shen-enOtremba, ZbigniewChen, Shih-JuiOtsuka, YuichiChen, Shiuan-YehOu, JianzhenChen, ShizhanOu, Jun-YuChen, ShuisenOu, ShumaoChen, ShuyanOu, Ting-ChiaChen, SongyueOussalah, MouradChen, TaoOuyang, BingChen, WeiOvando-Martínez, MaribelChen, XianfengØvsthus, KnutChen, XuexueOwens, Róisín M.Chen, YangquanOyekan, JohnChen, Yen-DaOyoshi, KeiChen, Yi-ChaoOzdemir, AnilChen, YiyunOzdemir, SahinChen, YongyaoOzen, Mehmet OzgunChen, Yuan-LiuOzer, EkinChen, Yuan-TsungOzeri, ShaulChen, Yu-ChangOzevin, DidemChen, YueOzgokman, TamayChen, Yu-HsuanP. Petropoulos, GeorgeChen, Yung-YuPaal, StephanieChen, YunpengPaavola, MarkoChen, YuweiPacchierotti, ClaudioChen, ZhenPace, PasqualeChen, ZhenghuaPacheco, JavierCheneler, DavidPacheco, João G.Cheng, ArthurPaci, FedericaCheng, Chia-HsinPadalkar, SonalCheng, Chiang-hoPaek, JeongyeupCheng, Chien-FuPaek, JungwookCheng, Chih-hsiuPage, AlexCheng, HongjuPage, WyattCheng, JingyuanPagès-Zamora, AlbaCheng, Kuang-WeiPaglialonga, AlessiaCheng, QiangPagliari, DianaCheng, Qingsha SPagnotta, LeonardoCheng, Shu-HuaPaik, Ho JungCheng, Wei ChenPaindavoine, MichelCheng, WeimingPaiva, SaraCheng, WeiyingPajovic, MilutinCheng, Wood-hiPak, DohyunCheng, XueminPak, On ShunCheng, YongzhiPakniyat, AliCheng, YuanPakrashi, VikramCheng, ZhiqiangPal, AmitangshuCheremkhin, PavelPalacios-Laloy, AgustinChernbumroong, SaisakulPalamartchouk, KirillChew, Zheng JunPaláncz, BélaChi, Yung-WeiPalaniappan, SubramanianChiabrando, FilibertoPalanisamy, SelvakumarChiadò, AlessandroPalazzo, SimoneChiang, Chen-KuoPalchetti, IlariaChiang, Chia-ChinPalego, CristianoChiang, JungshengPalella, Boris IgorChiang, Wei-HungPalermo, EduardoChiang, Yeo-WanPalermo, SamChiang, Yu-ChunPallaoro, AlessiaChiavaioli, FrancescoPalli, GianlucaChiavetta, UgoPalmerini, Giovanni B.Chiementin, XavierPalmerini, LucaChien, HwaPalomares, Jose ManuelChien, Jun-ChauPaloscia, SimonettaChien, Ying-RenPalumbo, FilippoChilds, PeterPalus, HenrykChiles, JeffPalzer, StefanChilo, JoséPamplona Segundo, MaurícioChilvery, AshwithPan, Guo-HuiChin, Bryan A.Pan, GuoqingChiolerio, AlessandroPan, Huang HsingChiriacò, Maria SerenaPan, LijiaChiu, Nan-FuPan, Tung-MingChizari, HassanPan, W. DavidCho, Dongil DanPan, YaozhongCho, Eun JeongPan, YunCho, GihwanPan, ZhigangCho, Gyou-JinPan, ZhongqiCho, HansangPanagakis, YannisCho, HohyunPanagiotakis, CostasCho, Ho-ShinPanagiotakis, SpyrosCho, Hsin-HungPanagopoulos, ThomasCho, JeonghunPanayides, Andreas S.Cho, Jin-HoPanda, ManojCho, SungboPandey, GauravCho, SungraePandey, RishikeshCho, Young ImPandolfi, AnnaChodak, MarcinPang, Da-ChenChodavarapu, VamsyPang, HongfengChohan, Balwant S.Pang, JunChoi (Prof.), AnthonyPang, ShintaroChoi, Byong-DeokPang, XiaobingChoi, Cheon WonPang, YoonsooChoi, Dong-HoonPangaluru, KishoreChoi, HeungjaePani, DaniloChoi, Hyun-HoPänkäälä, MikkoChoi, InheePanneton, BernardChoi, In-SikPanousopoulou, AthanasiaChoi, Jae WeonPantano, MariaChoi, Jae-WonPantazi, Xanthoula EiriniChoi, Jeong-WooPantazis, GeorgeChoi, Ji-WoongPaoletti, PaoloChoi, JongmooPapadopoulos, Georgios Z.Choi, KyoungahPapaelias, MayorkinosChoi, KyusunPapageorgas, PanagiotisChoi, Min-HyungPapaharalabos, GeorgeChoi, Sei-BumPapaioannou, ThanasisChoi, SeokheunPapanikolaou, VassilisChoi, Seon-JinPapoutsidakis, Michail G.Choi, SunwoongPappalardo, LucaChoi, Woo JunePappalettere, CarmineChoi, YongkiPappu, ChandraChong, AlvinParadells-Aspas, JosepChoo, JaebumParadiso, ValentinoChoo, RaymondParaforos, Dimitris S.Chopra, ShauhratParamelle, DavidChortos, AlexParaskevopoulou, SivyllaChou, Jue-SamParedes, FerranChou, Jung-ChuanParekh, SapunChou, Tien-YinParida, BikashChou, WushengPark, ByungwoonChow, Chi-WaiPark, Cheol-SooChow, Kin KeePark, DongheeChowdhury, EhsanPark, EdwardChowdhury, SagarPark, HyunjinChristaras, BasilePark, JaehyunChristensen, Jørn B.Park, Jee WoongChristiansson, Anna-KarinPark, JongwoongChrpa, LukasPark, Joon-ShikChryssomallis, Michael T.Park, JoontaekChu, Cheng-ShanePark, Jung-DongChu, JinkuiPark, JunyongChu, Shu-ChuanPark, Kang RyoungChu, TianxingPark, KwandongChua, Chun KiangPark, Kyoung YoulChuang, Cheng-hungPark, Kyung-JoonChuang, Kuo-ChihPark, SehyunChuang, MichaelPark, SeungheeChuang, Yen-JunPark, Soon-yongChuang, Yung-ChungPark, SuhyunChui, Hsiang-ChenPark, Suk-HeeChulhai, Dhabih VPark, UnsangChun, SejongPark, Yong-JaiChung, Chun-JenParker, BethCHUNG, SUNGWONParker, RichardChung, Yao-LiangParmehr, Ebadat G.Chung, Ying-ChienParolo, ClaudioCiabattoni, LucioParra, LorenaCiaccheri, LeonardoParra, VicenteCialla-May, DanaParrein, BenoîtCianca, ErnestinaParrilla, LuisCicchetti, AntonioParry, DaveCicchetti, RenatoPartsinevelos, PanagiotisCiecholewski, MarcinParvis, MarcoCieplak, MaciejPascoa, JoseCieslak, JérômePascucci, SimoneCięszczyk, SławomirPasian, MarcoCifuentes G., Carlos AndrésPasinszki, TiborCigna, FrancescaPasluosta, CristianCikajlo, ImrePasquali, PaoloCimalla, VolkerPasquier, ThomasCimellaro, Gian P.Passafiume, MarcoCiminelli, CaterinaPasserone, RobertoCindemir, UmutPassian, AliCinque, MarcelloPassilly, NicolasCinti, StefanoPassos, DiegoCionca, VictorPastore, RobertoCiosek, PatrycjaPata, PetrČiplys, DaumantasPatanè, FabrizioCipriani, ErnestoPatel, SaroshCirillo, AndreaPatil, DevendraCiuonzo, DomenicoPatimisco, PietroClaesen, JanPatricio, JorgeClaramunt, SergiPatrick, ErinClark, Adrian F.Patrikakis, CharalamposClark, ChristopherPau, GiovanniClark, LeonPaul, AnandClarke, AlastairPaulen, RadoslavClaudel, Christian G.Paulis, Francesco DeClausmeyer, JanPaulus, MarkClément, AlainPauwels, ValentijnClevers, JanPavanello, FabioClifford, Gari D.Pavliček, PavelCliment, Miguel ÁngelPavlichenko, IdaClimente-Alarcon, VicentePavlidis, IoannisCloninger, AlexanderPawlak, MichałClosas, PauPawlak, RyszardClose, Dan M.Pawlowski, AndrzejCloskey, David McPawlowski, MichalCobo, AdolfoPaya, LuisCobos, MaximoPayne, Christopher JCoburn, Craig A.Pazderski, DariuszCocconcelli, MarcoPaz-González, AntonioCoelho, João PintoPaziewski, JacekCoelho, LuisPazzi, Richard W.Cohenour, CurtisPecorella, TommasoCointault, FrédéricPecori, RiccardoCojoc, GheorghePedersen, Christian FischerCollette, ChristophePedersini, FedericoCollin, JussiPedersoli, MarcoCollins, David J.Pedreiras, PauloCollombet, FrancisPedrero, MariaCollotta, MarioPeer, PeterColosimo, GabrielePeham, ChristianColuccia, AngeloPék, ZoltánComite, DavidePekin, DenizCommeau, FrankPelegri-Sebastia, JoséConan, DenisPelissier, MichaëlConca Parra, Andrés Pellenz, Marcelo EduardoConchon, EmmanuelPellet, ClaudeConcilio, AntonioPelletier, MathewConforti, MassimoPelta, DavidConforto, SilviaPeltokangas, MikkoCong, XiaoyingPeltoniemi, JouniConmy, RobynPemen, A.J.M.Conti, AndreaPena-Garcia, AntonioConti, MassimoPeng, DiContini, DavidePeng, JianContreras-Medina, LuisPeng, JingConvington, JamesPeng, LimeiCook, DianePeng, RuoguCoppedè, NicolaPeng, YangCorchado, JuanPeng, YuexingCorchia, LauraPeng, YuxinCorcoran, JosephPeng, ZhengCorcoran, PeterPeng, ZhiliCordoba, AlbertoPenhaker, MarekCorrigan, DamionPenne, RudiCorsaro, CarmeloPensieri, SaraCorsi, CristianaPenzel, ThomasCortés López, Juan CarlosPepe, AntonioCosar, SerhanPeppas, Kostas P.Coscetta, AgnesePeralta, DanielCoskun, AhmetPercannella, GennaroCoskun, BulutPereira, AlcidesCosoli, SimonePereira, AntonioCosta Garcia, AgustinPereira, EulaliaCosta, CorradoPereira, NunoCosta, DanielPereira, TâniaCosta, Gilson A.O.P.Perera, CharithCosta, ManuelPerera, KithsiriCosta, NunoPeres, António ManuelCostantino, DomenicaPeres, EmanuelCostantino, GianpieroPerevertov, OleksiyCostanzi, RiccardoPérez Navarro, AntoniCostanzo, AlessandraPérez Oria, JuanCostanzo, AntonioPerez, AlfredoCostanzo, SandraPerez-Canto, SalvadorCosta-Requena, JosePérez-Jiménez, RafaelCostas, VassilakisPérez-Ocón, FranciscoCota-Ruiz, JuanPerez-Ramirez, Carlos AndresCouceiro, RicardoPerez-Ruiz, ManuelCowardin, HeatherPerez-Uribe, AndresCoxson, Gregory EmmettPergoloni, StefanoCozzolino, DanielPerinpanayagam, SureshCragg, PeterPeron, RobertoCramer, MichaelPerpiña, XavierCrandall, Aaron S.Perrone, GuidoCrane, Barbara A.Perry, MarcusCraven, Michael P.Persano, AnnaCrea, SimonaPersaud, KrishnaCrespo, JonathanPersijn, StefanCretu, Ana-MariaPertuz, SaidCrick, ChristopherPeruzzi, AgneseCrippa, PaoloPesatori, AlessandroCristea, CeciliaPescaru, DanCristina, Ana MajarenaPescosolido, LoretoCristina, MalegoriPesquer Mayos, LluísCrocco, LorenzoPessin, GustavoCroce, Anna CletaPessoa, LuísCroenne, CharlesPetcu, DanaCrovetti, JamesPeter, MichaelCrovetti, Paolo StefanoPeter, SteffenCruz, Homero ToralPetereit, JankoCruz-Hernández, CésarPeters, KaraCsizmadia, SzilardPeters, Thomas M.Csősz, ÉvaPetersen, JanCtistis, GeorgiosPetersen, Mark R.Cueva, Román Alcides LaraPeterson, BirgitCui, F.S.Peterson, DanielCui, YaoyaoPeterson, Donald R.Cui, ZiqiangPetković, BojanaCulver, R. LeePetráková, Vladimíra Cumanan, KanapathippillaiPetrellis, NikosCummins, GerardPetrie, HelenCuriac, Daniel-IoanPetrila, IulianCurtis, AaronPetroccia, RobertoCurto, AriadnaPetrone, GiuseppeCürüklü, BaranPetrov, DmitryCyganek, BoguslawPetrova, Krasimira T.Czajewski, WitoldPetsa, ElliCzech, PiotrPeveler, WilliamCzerwinski, DariuszPeyman, Sally A.D Lemaire, EdwardPeyrache, AdrienD’Addona, Doriana M.Peyton Jones, James C.Da Paixão, Thiago Regis Longo CesarPezza, LeonardoDa Ronch, AndreaPfeffer, JuliaDa Silva Cruz, Luis AlbertoPfeiffer, ThiesDa Silva, Joaquim C.G.E.Pfost, MartinDa Silva, José Machado Pham, Minh-TanDabove, PaoloPHAM, Quoc-VietDabrowski, JerzyPhan, Hoang-PhuongDacarro, GiacomoPhan, Manh-HuongDackermann, UlrikePhang, Swee KingDaems, DevinPhilippides, AndrewDagdeviren, CananPhillips, MarkDagenais, Michel R.Phinyomark, AngkoonDagiuklas, TasosPi, YimingDaher, NaseemPianini, DaniloDahlin, AndreasPiano, MartinaDai, Henry Hong-NingPiano, SamantaDai, RuiPiao, XianjiDai, XianmingPiao, YunxianDai, YuchaoPiasecki, TomaszDaidzic, NihadPicazo-Sanchez, PabloD'Alessandro, AntonellaPicerno, PietroD'Alimonte, DavidePieczonka, LukaszDaliri, AliPieper, JeffDalla Mora, AlbertoPieri, FrancescoDalla Vedova, Matteo D. L.Pierleoni, PaolaDallas, TimPifferi, FabienDaly, IanPigatto, Daniel FernandoDamas, MiguelPignaton De Freitas, EdisonDan, GuoPilehvar, SanazDán, GyörgyPillai, PrashantDandois, JonathanPillai, Premlal BalakrishnanD'andrea, CristianoPilloni, VirginiaD'Andreagiovanni, FabioPimenta Monteiro, MiguelDanescu, Radu GabrielPimenta, Tales CleberDang, VinhPinciroli, CarloDaniela, CarrionPinhasi, YosefDanielli, AmosPinho, PedroDansereau, Richard M.Pinilla-Cienfuegos, ElenaDanson, MarkPinilla-Gil, EduardoDantcheva, AntitzaPiotrowski, ZbigniewDantu, KarthikPiraino, FrancescoDao, Tien TuanPiras, AndreaDapo, AlminPires, GabrielDarafsheh, ArashPirinen, PekkaDardanelli, GinoPirkl, Ryan J.Darmon, MichelPiro, BenoitDarrozes, JoséPirozzi, SalvatoreDarsena, DonatellaPirri, Candido FabrizioDarvin, MaximPisani, MarcoDarwish, NasserPisco, MarcoDas, Anshuman J.Pisello, Anna LauraDas, AruneemaPiskozub, JacekDas, JagotamoyPislaru-Danescu, LucianDas, ManoharPissadakis, StavrosDatta, RupsaPissaloux, Edwige E.Datta, SambitPitchappa, PrakashDauphin-Ducharme, PhilippePitla, SantoshDavalli, AngeloPittella, ErikaDave, BhargavPiyare, RajeevDavid Hernández, JuanPizzolato, ClaudioDavid, SergioPlacet, VincentDavila, Juan CarlosPłaczek, BartłomiejDavis, Jason J.Płaczek, MarekDawn, ArnabPlasqui, GuyDay, Stephanie S.Platil, AntoninDe Angelis, AlessioPletl, SzilveszterDe Angelis, GuidoPlets, DavidDe Blasio, GabrielPlopski, AlexanderDe Bruin, SytzePnevmatikos, Nikos De Castro Megías, Ana IsabelPo, Lai-ManDe Cos Juez, Francisco JavierPochiraju, Kishore V.De Deyn, Gerlinde B.Podobnikar, TomažDe Diego, Isaac MartínPodržaj, PrimožDe Grande, RobsonPoisel, HansDe Jesus Pereira, António ManuelPolak, LadislavDe La Cruz Llopis, Luis J.Polavarapu, LakshminarayanaDe La Escosura, AlfredoPoletkin, KirillDe La Fuente, Jesús GrajalPoli, NicolaDe La Rica, RobertoPolichetti, TizianaDe La Torre, ÁngelPolikovsky, SenyaDe Lima Jr., Mauricio M.Poliks, MarkDe Luca, AndreaPolo, JoséDe Lucena, Sidney C.Polo-Parada, LuisDe Marchi, LucaPolrolniczak, EdwardDe Marcos Ruiz, SusanaPomante, LuigiDe Meo, PasqualePomorski, DenisDe Oliveira Albuquerque, RobsonPoncela, JavierDe Oliveira Barra, Guilherme MarizPonchak, George E.De Oliveira Moraes, AlisonPons, AlexanderDe Oliveira, Hélder Filipe PintoPontonnier, CharlesDe Pascali, ChiaraPooranian, ZahraDe Pasquale, ClaudioPop, Aniela I.De Paz, Juan F.Popa, Bogdan IoanDe Poorter, EliPopescu, DanDe Sanctis, MauroPopescu, MihailDe Simone, Salvatore GiovanniPopoola, OlalekanDe Souza, Éfren LopesPoria, SoujanyaDe Stefano, LucaPořízka, PavelDe Vasconcelos, Elder AlpesPorras-Aguilar, RosarioDe Vito, SaverioPorter, Daniel AllenDe, SuparnaPortilla, Jorge HigueraDean, KevinPoslad, StefanDean, RobertPostigo, Pablo AitorDebela, Ahmed MehdiPotamitis, IlyasDedu, EugenPotma, EricDehais, JoachimPotortì, FrancescoDehaut, AlexandrePotter, John RDehghanian, VahidPoulakis, NikolaosDehghan-Niri, EhsanPound, MichaelDel Campo, Francisco JavierPourhomayoun, MohammadDel Din, SilviaPourreza, AlirezaDel Rio Celestino, MercedesPowell, RebeccaDel Rosal, BlancaPowell, SamuelDel Villar, IgnacioPoza-Lujan, Jose-LuisDelabie, TjorvenPozo, FrancescDelaney, Declan T.Pozza, RiccardoDelashmit, WalterPozzebon, AlessandroDelchev, KamenPozzi, FedericoDeleforge, AntoinePrabhu, RadhakrishnaDelepine-Lesoille, SylviePrabhulkar, ShradhaDelgado Prieto, MiguelPradalier, CédricDelgado, JaimePradhan, NiharDelgado, JorgePrager, JensDelgado-Gonzalo, RicardPrakash, RanganathanDelhaye, BenoitPramudianto, FerryDeligianni, FaniPrandi, CatiaDelis, AlexPranggono, BernardiDelis, PaulinaPrasad, Dilip K.Dell’Orco, MauroPrasad, GauthamDella Corte, FrancescoPrasser, Horst-MichaelDell'Aversano, AngelaPratsinis, Sotiris E.Dell'Olio, FrancescoPreciado, Miguel ADelmas, Jean-PierrePrecup, Radu-EmilDelmotte, FrançoisPredoi, Mihai V.Del-Val, LaraPreece, AlanDemestichas, KonstantinosPremebida, CristianoDeMiguel-Ramos, MarioPrentkovskis, OlegasDemircan, EmelPresas, AlexandreDEMIRCI, IbrahimPresti, Domenico LoDemo Souza, RichardPreußler, StefanDen, WalterPrezioso, GiuseppinaDeng, HuaPřibilová, AnnaDeng, MingcongPrice, Owen F.Deng, YingbinPrimak, SergueiDeng, ZhiguoPrior, Stephen D.Deng, ZhiHongProcek, MarcinDenis, KathleenProchazka, AlesDeo, RandhirProchazka, DavidDeschinkel, KarineProtopapadakis, EftychiosDeshpande, NikhilProulx, Michael J.Deshpande, SagarPrudenzano, FrancescoDesmulliez, MarcPruneanu, StelaDeSouza, GuilhermePryss, RüdigerDestelle, FrancoisPrzyborski, MarekDestercke, SébastienPrzybyla, Richard J.Dettmering, DenisePrzybyt, MałgorzataDev, SoumyabrataPrzysowa, RadoslawDevadas, Mary SajiniPsannis, KonstantinosDevkota, JagannathPu, HaihuiDevries, K.L.Pu, ShengliDeVries, LeviPudelko, RafalDey, SatadruPugi, LucaDhakal, AshimPuig, VicencDhakal, SagarPuiu, MihaelaDhaou, RiadhPujo, Mariacinta C.Di Antonio, MarcoPujol Nadal, RamonDi Bartolomeo, AntonioPulaski, ŁukaszDi Benedetti, MatteoPuletti, NicolaDi Camillo, BarbaraPullano, SalvatoreDi Capua, R.Pursley, BrennanDi Martino, GerardoPustan, MariusDi Martire, DiegoPutilov, Arcady A.Di Massa, GiandomenicoPuton, JarosławDi Pasquale, FabrizioPyk, PawelDi Ruberto, CeciliaPyrchla, JerzyDiamond, DermotQamar, AfzaalDianat, PouyaQasaimeh, MohammadDias Da Costa, DanielQi, HengDias Da Cunha, RudneiQian, GangDias, JorgeQian, JiuchaoDias, LuisQian, ZhenyunDias, Mary BernardineQiao, GangDias, PauloQiao, YuansongDíaz Fernández, BelénQiao, ZhijunDíaz Navas, José AntonioQin, HantangDíaz-Cruz, José ManuelQin, PeiyuanDickens, TarikQin, YongDickert, Franz L.Qin, YongruiDieber, BernhardQin, ZhengruiDiem, MarkusQiu, TieDierickx, BartQiu, XianboDiessel, OliverQiu, XiaoqunDiethe, TomQiu, YongDietzel, StefanQiu, YongqiangDíez, Isabel De La Torre Qu, FengzhongDight, PhilQu, HongweiDikaios, NikolaosQuattrini Li, AlbertoDillon, DanielQueirós, AlexandraDilo, ArtaQueirós, SandroDilonardo, ElenaQueiruga-Dios, AraceliDimitrios, KogiasQuer, StefanoDinca, Lavinia MihaelaQuero, GiuseppeDincer, CanQuinn, Mark KennethDing, GuoruQuintana, Penelope.J.E. Ding, Jow-LianQuitadamo, LuciaDing, MengRaahemifar, KaamranDing, XiaorongRabadan, JoseDing, XinghaoRabbani, Muhammad SaqibDing, Yin (Adam)Rabbou, Mahmoud AbdDing, YongRabczuk, TimonDing, YueminRabia, JafriDing, ZhifengRabin, NetaDinh, ToanRackauskas, SimasDinis, RuiRad, CiprianDiouris, Jean-FrançoisRadamson, Henry HDiraco, GiovanniRadecka, HannaDixit, Chandra K.Radecki, JerzyDixon, CoryRadhakrishnan, SivaprakasamDixon, SteveRadu, Gabriel-LucianDjeziri, MohandRadwan, AymanDjuric, NikolaRadzieński, MaciejD'Lima, DarrylRae, ColinDo, Phu ThinhRagulskis, MinvydasDobbins, ChelseaRahat, Alma A. M.Dobler, GregoryRahbar, KatayounDobosz, MarekRaheja, AmarDobrokhotov, VladimirRahimabady, MojtabaDoeven, EganRahman, Mir MustafizurDogan, AtillaRahman, MusfiqDoh, NakjuRahul, KishorDoktor, DanielRai, PratyushDolbeau, RomainRaimondi, Francesco MariaDomaneschi, MarcoRains, GlenDomaszewicz, JarosławRajan, GinuDomenico, GrimaldiRajic, NikDomingues, FátimaRakhshan, AliDominguez Brito, Antonio CarlosRakovic, MirkoDominguez-Pumar, ManuelRakun, JurijDominici, DonatellaRamadan, QasemDOMINO, Krzysztof A.Ramakrishnan, Arun KishoreDonarelli, MaurizioRamalingam, RajinikumarDonati, LorenzoRamanathan, RajeshDonati, SilvanoRamanavicius, ArunasDonelli, MassimoRamapriyan, HampapuramDong, HaiRamasamy, SubramanianDong, HefengRamasso, EmmanuelDong, LeiRamaswamy, PalaniappanDong, TaifengRamella, GiulianaDong, TaoRamezani, MohsenDong, WenRamirez Miquet, EvelioDong, XiuzhenRamírez Rivera, AdínDong, YongkangRamirez-Paredes, Juan PabloDong, YouRamirez-Vick, JaimeDong, ZhenRamón Fabresse, FelipeDoni, Mihaela BadeaRamondini, MassimoDonlagic, DenisRamos, VictorDonnell, Kristen M.Ramos, VictoriaDonoso, YezidRamos-Ortiz, GabrielDonzella, ValentinaRampa, VittorioDoran, DerekRampazzi, Sarad'Orey, PedroRamsey, Ian ScottDoria, AlbertoRamsköld, DanielDormido Bencomo, SebastianRan, GuangzhaoDorronzoro, EnriqueRana, DipakDorsey, Kristen L.Ranganathan, AanjhanDorsinville, RogerRangelova, ElenaDos Santos, DanielRantanen, VilleDos Santos, SergeRao, AravindaDosen, StrahinjaRao, Posinasetti NageswaraDoubell, MarkRao, QinchunDoulamis, AnastasiosRao, SmithaDoulamis, Nikolaos D.Raparelli, TerenzianoDovis, FabioRaposo, MariaDowrick, ThomasRapp, MichaelDowsley, RafaelRapson, Trevor D.Dragic, PeterRaptis, TheofanisDrap, PierreRäsänen, AleksiDrapikowski, PawełRascher, RolfDrexler, Petr Rascon, CalebDrost, BertramRashedi, EhsanDrusa, MariánRasmussen, Morten DamDu Plessis, WarrenRathore, SaimaDu, JianRatilal, PurnimaDu, JianhaoRaul Marin, EngDu, LanRaun, WilliamDu, WanRauschnabel, Philipp A.Du, Yi-ChunRaut, SangramDu, ZhenhuiRavankar, AbhijeetDualibe, Fortunato C.Ravankar, AnkitDuan, HanchenRavelo, BlaiseDuan, JinmingRavera, EnricoDuan, QiangRawassizadeh, RezaDuan, WeiliRawat, PriyankaDuan, XuexinRawstorn, Jonathan C.Duan, YucongRazaque, AbdulDuann, Jeng-RenRazevig, VladimirDuarte, CarlosRazionale, Armando VivianoDubach, John MatthewRdzanek, Wojciech P.Dubbelman, GijsRe, RebeccaDubey, AbhishekRea, PierluigiDubey, HarishchandraRebaudengo, MaurizioDubuc, DavidRebecchi, FilippoDucati, Jorge RicardoReboul, SergeDuczek, SaschaRedding, BrandonDuda, Timothy F.Redweik, PaulaDudczyk, JanuszReed, NickDufresne, AnjaReenalda, JasperDuguay, YannickReeves, Daniel BDuma, LuminitaRegier, MarcDuma, Virgil-FlorinRegmi, BishnuDumee, LudovicRegmi, Bishnu P.Dumitru, Andra-CristinaRego, GasparDumitru, LiviuRegulski, KrzysztofDumoulin, CédricRehmani, Mubashir HusainDunai, LarisaReig, CandidDuncan, Alec JReimann, JensDungan, Jennifer L.Reimhult, ErikDunik, JindrichReina, AndreagiovanniDuong, TrungReina, Daniel GutiérrezDuquennoy, MarcReina, GiulioDuque-Perez, OscarReindl, LeonhardDuraia, El-shazly M.Reineck, PhilippDurán Acevedo, Cristhian ManuelReinhardt, AlexandreDuro, NatividadReinoso, O.Dutta, AnirbanReinoso, OscarDutton, NealeReinsch, ThomasDuy, Le ThaiReis, ArsénioDvoyrin, VladislavReis, Manuel J.C.S.Dworak, VolkerReis, NunoDyo, VladimirReisenfeld, SamDzantiev, BorisReising, Donald R.Dziuda, ŁukaszReiter, AustinDziurdzia, PiotrReljin, NatasaDzvonkovskaya, AnnaRemillieux, MarcelE Silva, Francisco José Da Silva Ren, JianfengE. Tripoliti, EvanthiaRen, JinchangEbeid, Hala M.Ren, KangningEbrahimi, AmirRen, MingjunEbrahimkhanlou, ArvinRen, PinyiEbralidze, Iraklii I. Ren, WeiEcheto, Francesc MollRen, XiaojunEchtermeyer, TimRen, YiEckert, MartinaRen, YongEconomou, AnastasiosRenard, Jean-BaptisteEdirisinghe, MohanRendón Mancha, Juan ManuelEdwan, EzzaldeenRenevey, PhilippeEdwards, AlistairRengarajan, RajagopalanEdwards, Rachel S.Renso, ChiaraEftekhar Azam, SaeedRenzoni, FerruccioEfthymoglou, George P.Reponen, JarmoEgawa, YuyaResano, JavierEiza, Mahmoud HashemResconi, Virginia CeliaEklundh, LarsRestuccia, FrancescoEkstrom, MikaelRetscher, GüntherEl Ali, AbdallahRevaprasadu, NeerishElad, TalReverter, FerranElbahhar, Fouzia BoukourRevuru, Rukmini SrikantElbaum, RivkaRey, GaëtanEldhuset, KnutReyes, Pablo AndresEleftherakis, GeorgeReyes, Pavel IvanoffElgendi, MohamedRhee, HuinamElhabian, Shireen Y.Rho, JunsukEliasson, JensRhudy, MatthewEliopoulos, Panagiotis A.Riahi, RezaElMoslimany, AhmadRiaud, AntoineEl-Osery, Aly I.Riaz, QaiserEl-Sankary, KamalRibeiro, AngelaElsts, AtisRibeiro, Artur LopesEltoukhy, MoatazRibeiro, MoisesEmadi, ArezooRibo Vedrilla, SerniEmmanuele, Andrea Riboni, DanieleEmoto, TakahiroRicci, MarcoEndo, TatsuroRicci, StefanoEndres, DominikRice, GraceEngelbrecht, RainerRice, Matt ThomasEngelhard, MatthewRichiedei, DarioEngels, DanielRichtera, LukášEngemann, DenisRickert, MarkusEngland, AndrewRicketts, David S.Englebienne, GwennRiedl, MatthiasEnguita, José María Rigoni, FedericaEnnen, IngaRiguidel, MichelEnpuku, KeijiRiley, BryanEnright, JohnRim, You SeungEnrriquez, Haggeo DesirenaRinaldi, FabioEnser, HerbertRinaldi, StefanoErenas, Miguel MaríaRinaldo, RobertoErives, HectorRinkeviciene, RomaErmagun, AlirezaRinzivillo, SalvatoreErnst, JosephRiordan, DanielErny, Guillaume L.Rios, RubenErra, UgoRioual, S.Ersen, AliRipka, PavelErsoy, IlkerRipken, TammoEscribano, Javier GómezRisoluti, RobertaEsffahani, AlirezaRitcey, JamesEshagh, MehdiRivadeneyra, AlmudenaEskina, Erika NRivadeneyra-Torres, AlmudenaEspinosa, JuliánRivnay, JonathanEspinosa-Urgel, ManuelRiziotis, ChristosEsposito, ChristianRizzardi, AlessandraEsteban, SegundoRizzi, FrancescoEsteban-Fernández De Ávila, BertaRizzini, Dario LodiEstevez, FranciscoRizzo, PiervincenzoEstrela, PedroRizzolo, SerenaEstrela, Vania VieiraRo, RuyenEstrella, Júlio CezarRo, Seung-KookEtemadi, MozziyarRoatta, SilvestroEtienne, DagueRobardet, CelineEtzlinger, BernhardRobert, JörgEuler, William B.Roberts, CraigEusebio, LidiaRobertson, William M.Evans, Mathew H.Robin, François B.Evtugyn, GennadyRobinson, John A.Ewing, MarkRobles, GuillermoEwing, TimothyRobles, RamiroExall, KirstenRocca, FabioFabbri, BarbaraRocco, FurferiFabbrocino, GiovanniRocha, Luís A.Faber, GertRocheleau, TristanFabian, MatthiasRoda, AldoFabregas, ErnestoRodenas-Herráiz, DavidFadda, MauroRodrigues, EduardoFaia, Pedro Manuel Gens Azevedo MatosRodrigues, Joel J. P. C.Faigl, JanRodrigues, José MiguelFaiña, AndresRodríguez Ángeles, AlejandroFaion, FlorianRodríguez Martin, DanielFakiris, EliasRodríguez Méndez, María LuzFalchi, FabrizioRodríguez Polo, ÓscarFalciola, LuigiRodriguez, DionisioFalco, GianlucaRodríguez, Teresa PinedaFalconi, Marta TeclaRodriguez-Manzano, JesusFamaey, JeroenRodríguez-Martín, ManuelFan, BinRodriguez-Pino, MarcosFan, KangqiRodríguez-Pulido, Francisco J.Fan, LeiRodriguez-Resendiz, JuvenalFan, WeihongRoerdink, MelvynFan, ZhaoyanRogier, FrancoisFan, ZhengRogier, HendrikFang, FeiRohac, JanFang, LeiRoig, IgnacioFang, LuRoj, JerzyFang, PengRomaniello, RobertoFang, RonnieRomero-Troncoso, Prof. Rene de JesusFang, Shih-HauRomine, WilliamFang, XingRönnholm, PetriFang, XudongRopokis, George A.Fang, YiRoriz, PauloFang, YihaiRos Lis, Jose VicenteFang, YinfengRos, GerardFantozzi, SilviaRosa, NelsonFaragasso, AngelaRosa, StefanoFarahbakhsh, RezaRosario, DenisFaria, Diego R.Roscia, Maria CristinaFarias, GonzaloRose, Joseph L.Farinella, GiovanniRosen, LarsFarlik, JanRosen, ShaleFarooq, MuhammadRosenberg, LukeFarrokhrooz, MehdiRosenberger, A.T.Farroni, FlavioRosi, AscanioFaruque, Imraan A.Rosique Contreras, María Francisca Fasana, AlessandroRosolem, Joao BatistaFasano, AndreaRosolino, VaianaFasano, GiancarmineRossella, FrancescoFasel, BenediktRossi, MicheleFassi, FrancescoRossi, René M.Fauss, MichaelRossi, StefanoFavero, Simone DelRossignol, JérômeFayaz, MohammadrezaRossomando, FranciscoFazio, PeppinoRotariu, LucianFedorko, GabrielRoth, BernhardFeiertag, GregorRoth, GunterFeixas, Francesc SebéRoth, KeelyFekete, ZoltánRothkrantz, LeonFeldens, PeterRottondi, CristinaFelicetti, LucaRouillard, JoséFelici-Castell, SantiagoRousseau, DavidFelipe Oliveira, SilvaRoussey, CatherineFelis, IvanRovira, AitorFelisberto, PauloRowlandson, TracyFelix Navarro, KarlaRoy, AditiFelix, Anderson AndréRoy, SurajitFeller, Jean-FrancoisRoyo, SantiagoFelski, AndrzejRozado, DavidFemminella, MauroRozenstein, OfferFeng, BoningRozga, PawelFeng, ChuangRuan, HaihuiFeng, DongmingRuan, HaowenFeng, JianRuano Garcia, Maria VictoriaFeng, JingRudnitskaya, AlisaFeng, RuiminRuffaldi, EmanueleFeng, ShaojunRuffin, PaulFeng, XianyongRufino, GiancarloFenoglio-Marc, LucianaRühmer, DennisFentanes, Jaime PulidoRuijters, DanielFerdyn-Grygierek, JoannaRuiz Barquín, RobertoFereidountabar, AmirhosseinRuiz Llata, MartaFerguson-Pell, MartinRuiz-Hincapie, PaulaFerhan, Abdul RahimRumyantseva, MarinaFernandes, ArmandoRuotsalainen, LauraFernandes, HenriqueRupi, FedericoFernandes, TelmoRussell, ChristopherFernández Berni, JorgeRusso, PaoloFernández Caballero, AntonioRusu, CristinaFernández Caramés, Tiago M.Ryan, ASFernández Díez, María JesúsRyan, MichaelFernández Ramos, María DoloresRydosz, ArturFernández Romero, Juan ManuelRyu, DonghyeonFERNÁNDEZ, ANTONIORyu, SangjinFernández, Óscar BelmonteRyżak, MagdalenaFernandez, RoemiRządkowski, RomualdFernández, SuselRzhanov, YuriFernandez-Garcia, RaulRzonca, DariuszFernandez-Hernandez, JesusSa, InkyuFernandez-Llatas, CarlosSaadat, MozafarFernandez-Lopez, HelenaSaadeh, MohammadFernando, NuwanthaSaal, HannesFernando, XavierSaavedra, Mario LilloFerracuti, FrancescoSabatini, RobertoFerrández-Pastor, Francisco JavierSaberioon, MohammadmehdiFerrarezi, RhuanitoSabo, ChelseaFerrari, CarloSabri, YliasFerrari, GianluigiSacré, Pierre-YvesFerraro, PietroSadeghi, Mahdi MFerraz, CarlosSadjadi, FiroozFerrein, AlexanderSadowski, LukaszFerreira, ArmandoSadre, RaminFerreira, HugoSaeed, KhalidFerreira, João Carlos AmaroSaez De Adana, FranciscoFerreira, José VasconcelosSafacas, AthanasiosFerreira, NivanŠafarič, RikoFerrer Crespo, Maria BelenSagduyu, Yalin EvrenFerrero, RenatoSagebiel, John C.Ferrero, SergioSagers, JasonFerrigno, LuigiSaggio, GiovanniFerro, MarcelloSaharan, LalitaFerruzzi, GabriellaSahay, PeeyushFicco, MassimoSahidullah, MdFiglus, TomaszSahoo, JagrutiFigueiras, EditeSahoo, Prasan KumarFigueiredo, LinoSahoo, Sujit KumarFigueroa, MiguelSahore, VishalFigurski, MariuszSahuquillo Balbuena, ElviraFilippeschi, AlessandroSainidou, RebeccaFilippoupolitis, AvgoustinosSainz, Beatriz Filleter, TobinSainz, Nekane IoneFingas, MervSaitoh, TakeshiFink, Glenn A.Sakamoto, João Marcos SalviFino, PeterSakamoto, TakuyaFioranelli, FrancescoSakr, Ahmed HamdiFiorani, AndreaSakumura, YuichiFiore, MarcoSalach, JacekFiorini, LauraSalamí, EstherFiroozy, NarimanSalanova Grau, Josep MariaFirouzi, MahshidSalau, J.Fischer, GeorgSalaun, PascalFischer, Wolf-Joachim J.Salazar, AddissonFischerauer, GerhardSalazar-Torres, Jose De JesusFisher, AdrianSalberg, Arnt-BørreFlechsig, Gerd-UweSales, JorgeFleisher, Adam J.Sales, SalvadorFleming, AaronSalgueiro, José RamónFletcher, AndrewSalm, CoraFletcher, Reginald S.Salmi, MikaFletcher, SimonSalowitz, NathanFletcher, Steven J.Salski, BartlomiejFleury, AnthonySaltogianni, VassoFlores, GerardoSalumäe, TaaviFlores-de-Santiago, FranciscoSalvi, DarioFlores-Tapia, DanielSalvo, PietroFoguel, Marcos ViniciusSamaras, IoannisFolea, SilviuSamba, RamonaFonollosa, JordiSamczynski, PiotrFonseca, Manuel J.Samiappan, SathishkumarFontecave, JulieSampaio, SilvioFontecha, JesúsSampangi, Raghav V.Foo, ErnestSampietro, DanieleFoong, ShaohuiSánchez De Rojas, JoseluisForczmanski, PawelSánchez, AlfredoForke, RomanSanchez, Jean-BaptisteForlani, GianfrancoSánchez, María-teresaForler, ChristianSanchez, NildaFormisano, AlessandroSánchez, Pablo Pedro Fornaser, AlbertoSánchez, René-VinicioForshaw, MatthewSanchez, VictorFörster, AnnaSánchez-Durán, Jose A.Forsyth, JasonSanchez-Gomez, JesusFort, AdaSanchez-Rodriguez, DavidFortes, SergioSanchiz, JoaquinFortino, GiancarloSand, JohanFortuna, LuigiSandasi, MaxleeneFortune, EmmaSanders, JamesFosson, SophieSandhu, AdarshFoster, AlecSandoval Orozco, Ana LucilaFoster, ChristopherSang, JizhangFotiadis, DimitriosSang, Tzu-HsienFotopoulos, GeorgiaSanguesa, JulioFouchal, HaceneSanguinetti, Joseph L.Foucher, SamuelSanislav, TeodoraFourati, NajlaSanli, AbdulkadirFourie, DehannSanMiguel, Juan C.Fowler, BoydSanphuang, VaritthaFox, GeoffreySan-Segundo, RubénFraddosio, AguinaldoSansone, LuciaFraga, Francisco J.Sant’ Anna, AnitaFraga-Lamas, PaulaSantamaria, EduardFragkiadakis, AlexandrosSantana, José MiguelFraile-Marinero, Juan-CarlosSantarato, GiovanniFrancalanza, EmmanuelSante, Raffaella DiFrancis, LaurentSanthanagopalan, SunandFranco, RicardoSanti, EmanueleFranco, WalterSanti, FabrizioFrangi, AttilioSantini, MaurizioFrank, RaphaelSantonico, MarcoFranke, Ingmar S.Santonicola, MariagabriellaFranke, JoergSantos, AbelFranks, AshleySantos, Davi AntônioFränti, PasiSantos, Frederico MiguelFrappart, FrédéricSantos, Jesús DanielFrasca, PaoloSantos, Juan M.Fraternali, PieroSantos, LídiaFrazzon, Enzo MorosiniSantos, PauloFreddi, AlessandroSantos, RodrigoFreire, DavidSantos, Telmo G.French, AndrewSantos-Assunção, SóniaFreschi, ValerioSantulli, CarloFrese, ChristianSanwar Hosen, A. S. M.Frey, MargaretSanyal, ArindamFreymueller, JeffSanz-Ablanedo, EnocFridman, LexSapienza, MichaelFridman, MotiSaponara, SergioFriedman, JasonSarfraz, JawadFriedrich, CraigSarigiannidis, PanagiotisFriedt, Jean-MichelSarioglu, A. FatihFrigieri, Edielson PrevatoSariyildiz, EmreFritsch, DieterSarkar, TapanFritz, Roschelle (Shelly)Sarrafzadeh, HosseinFritzsche, MarcoSarraguça, MafaldaFrizera, AnselmoSaruhan, BilgeFröhlich, ThomasSasagawa, KiyotakaFröjdh, ErikSasaki, EiichiFrontoni, EmanueleSasaki, KenFrueh, CarolinSasayama, TeruyoshiFryskowska, AnnaSatici, AykutFu, ChanghongSATO, HiroyukiFu, HongSatoh, HisashiFu, PengSatorres, Silvia MartínezFu, Yun RaymondSaukh, OlgaFuhrmann, ThomasSavagatrup, SucholFujinami, KaoriSaval-Calvo, MarceloFujioka, KoukiSavazzi, PietroFujiwara, EricSavi, PatriziaFujiwara, KoichiSavin, IgorFulginei, Francesco RigantiSavitski, DzmitryFumagalli, LucaSawacha, ZimiFung, Wai-keungSaxena, NavratiFunk, MarkusSazykin, StanislavFurutani, KatsushiSberveglieri, VeronicaFuse, TakashiScannapieco, Antonio FulvioFuso, FrancescoScappatici, LorenzoFutagawa, MasatoScarabelli, LeonardoGaboury, SebastienScarcia, UmbertoGachet Páez, DiegoSchabowicz, KrzysztofGaczynska, MariaSchaefer, Hans-EckhardtGade, MartinSchaefer, MickGade, RikkeSchall, MarkGaglione, SalvatoreSchauer, ThomasGai, KekeScheel, MichaelGaiani, MarcoScheer, Hella-ChristinGaillot, Davy P.Scheiber, RolfGaitas, AngeloScheller, FriederGajda, JanuszScheller, MaikGajos, KatarzynaSchena, EmilianoGalambos, TibiSchenato, LucaGalatus, RamonaSchiffman, SusanGalceran, ReginaSchilt, StephaneGalisteo Jr., Andres JimenezSchindelhauer, ChristianGallagher, John C.Schirhagl, RomanaGallagher, Justin FrancisSchirrmann, MichaelGalland, StéphaneSchlachetzki, JohannesGallay, MichalSchlögel, RomyGallego, AntolinoSchmeitz, AntoineGallego, Javier F.Schmidt, AdamGalleguillos, MauricioSchmidt, Mikołaj KajetanGallicchio, ClaudioSchmitt, CorinnaGalloway, Kenneth F.Schmitt, RobertGalmes, SebastiaSchmittmann, OliverGaloogahi, Hamed KianiSchmitz, AlexanderGalopin, NicolasSchmuker, MichaelGalstyan, VardanSchneider Von Deimling, JensGalvan-Tejada, Carlos EricSchneider, MichaelGambi, EnnioSchoedel, AlexanderGamboa, HugoScholefield, AdamGamo, JavierScholkmann, FelixGamse, SonjaScholl, IngridGan, LuScholten, KeeGan, NingScholz, MarkusGanapathy, SubhashiniSchönemann, LarsGandino, FilippoSchormans, JohnGanesan, BaskaranSchotter, JörgGanser, ChristianSchreier, GünterGanzfried, SamSchultz, StephenGao, BinSchulz, RuthGao, CaicaiSchulze Lammers, PeterGao, David (Zhiwei)Schumacher, JensGao, DongyueSchumann, FrankGao, HuijunSchumann, HenrikGao, LeiSchütze, AndreasGao, SongSchwartz, ChristopherGao, WeiluSchwartz, David EricGao, WenpeiSchwarz, StefanGao, XuanSchwenke, HeinrichGao, XuefeiSchwieger, VolkerGao, YanScida, KarenGao, YueScioscia, FlorianoGao, ZaifengScogings, ChrisGao, ZheScotney, BryanGao, ZhenScully, Christopher G.Gao, ZhiqiangSecco, Emanuele LindoGarbaras, AndriusSechidis, Lazaros A.Garbin, EnricoSeder, MarijaGarcia Breijo, EduardoSee, Chan H.García Sanchez, FelipeSeeger, ThomasGarcia Vazquez, Juan PabloSeel, ThomasGarcía, AntonioSeethaler, Rudolf J.García, Carmelo R.Segawa, HiroyoGarcía, Juan C.Seguin, FabriceGarcia, Miguel AngelSegura Garcia, JaumeGarcia, Nuno M.Segvic, SinisaGarcía, TaniaSeher, MatthiasGarcia-Baños, BeatrizSeitz, JochenGarcia-Cabot, AntonioSeitz, PeterGarcia-Ceja, EnriqueSeitz, W. RudolfGarcía-Chamizo, Juan ManuelSeixas, AdéritoGarcía-Fernández, Ángel F.Sekretaryova, AlinaGarcia-Fidalgo, EmilioSela, LinaGarcía-Martinez, Joaquín C.Selesnick, IvanGarcía-Miquel, H.Selker, JohnGarcia-Pedrero, AngelSelleri, StefanoGarcía-Pineda, MiguelSelvadurai, PaulGarcía-Rubio, CarlosSelvamani, AmuthaGarcia-Santos, VicenteSelyanchyn, RomanGarcia-Segura, SergiSemanjski, IvanaGarcia-Villegas, EduardSemenova, YuliyaGardi, AlessandroSemmar, NadjibGardiner, BryanSendra, SandraGardiner, PhilipSenesky, DebbieGarey, JamesSenetakis, KonstantinosGarg, AkhilSengupta, BidishaGargano, MarcoSenkal, DorukGargiulo, GaetanoSenthilnath, J.Gargiulo, Gaetano DSeo, Bo-SeokGarrido, RubénSeo, Dong-HoanGartia, ManasSeo, HwajeongGarzón, Gracia Ester MartínSeo, Min-WoongGasbarri, PaoloSeoane, FernandoGasch, CaleySeoane, LuciaGascon, HugoSeppa, Ville-PekkaGąska, AdamSepulveda, AbdonGasparini, LeonardoSequeira, AnaGašparović, MateoSequeira, JeanGastaldi, LauraSequeira, JoãoGastaldo, PaoloSerafino, FrancescoGates, ByronSergievskaya, I.Gatteschi, ValentinaSergiou, CharalambosGattulli, VincenzoSerio, CarmineGaudin, ValerieSerna, AndrésGautier, MatthieuSerôdio, CarlosGavazzi, BrunoSerrano, EmilioGavves, EfstratiosSerrano, José IgnacioGawanmeh, AmjadSerrao, SebastianoGay, MichelSerry, MohamedGbadebo, AdenowoSesay, AbuGe, XiaohuSesen, MuhsincanGe, XudongSettgast, VolkerGe, YufengSeverino, RicardoGebhardt-Henrich, Sabine G.Sfarra, StefanoGębicki, JacekSgora, AggelikiGeirsson, HalldorSha, KeweiGeissler, DanielShacham-Diamand, YosiGemeiner, PeterShafiei, MahnazGendron, PaulShafik, MahmoudGeneiatakis, DimitrisShafique, MuhammadGeng, JianghuiShah, BrualGeng, JunlongShah, PratikkumarGeng, YishuangShah, RashmiGenge, BélaShah, Syed AhmarGenis, VladimirShah, ViralGennarelli, GianlucaShahab, ShimaGenovese, AngeloShahbazi, MahmoudGenovesi, SimoneShahbazi, MozhdehGente, RalfShaheen, S. A.Gentili, Rodolphe J.Shahid, AdnanGeorgantas, NikolaosShahrabi-Farahani, AlirezaGeorgiadis, ApostolosShahrestani, SeyedGeorgiadis, CharalamposShaker, GeorgeGeorgiou, OrestisShakev, NikolaGeorgoulias, AristeidisShalev, GilGerbino, SalvatoreShan, MaoGerken, MartinaShan, Xiaojun (Gene)Geronimo, Andrew M.Shang, FangGerostathopoulos, IliasShao, DangdangGersen, HenkjanShao, PengGevorgian, SpartakShao, ZhenfengGhafar-Zadeh, EbrahimSharan, NekGhahramani, AliShariati, AliGhahremani, KasraSharma, BhavyaGhamari, MohammadSharma, DeeptiGharanjik, AhmadSharma, LakeshGharbaoui, MolkaSharma, MrigankGharechelou, SaeidSharma, Piyush SindhuGharehbaghi, ArashSharma, RajnikantGhavidel, AliSharma, Shree KrishnaGhayvat, HemantShartner, ErikGheorghiu, Razvan AndreiShemelya, CoreyGherlone, MarcoShemer, LevGhiasi, SoheilShen, ChangqingGhilain, NicolasShen, ChaoGhisi, AldoShen, Guo ZhenGhods, PouriaShen, JunGholamhosseini, HamidShen, LianfengGhommam, JawharShen, MeiGhose, DebasishShen, Yu-FangGhosh, AniruddhaShendryk, IuriiGhosh, ArijitSheng, DuoGhosh, DebashisSheng, WenyiGhosh, IndrajitSheppard, JonathanGhosh, SreyaSherratt, R SimonGiacobbe, MaurizioSheta, BassemGiacomini, MauroSheu, Meng-LiehGiagkos, AlexandrosShevtsov, Sergey N.Giakos, GeorgeShi, BoxinGiammarile, FrancescoShi, FanGiannakopoulos, TheodorosShi, JunboGiannelli, CarloShi, KeGiansanti, LuisaShi, LeiGiardina, PaolaShi, TiezhuGiasin, KhaledShi, XianmingGienko, GennadyShi, ZhenweiGiergiel, MariuszShi, ZhiguoGiesko, TomaszShiang, Tzyy-YuangGiffney, TimShiao, YaojungGiguere, PhilippeShibata, MizuhoGil, DavidShibata, TakayukiGil, EduardoShieh, Chin-ShiuhGil, Eric De SouzaShieh, Wann-YunGilbert, Hunter B.Shih, Ching-longGilbert, James M.Shih, Kuei-PingGillham, MichaelShih, Sheng-WenGillich, Gilbert-RainerShih, SteveGiner, JoanShih, Wen-PinGiner-Casares, Juan J.Shikida, MitsuhiroGini, GiuseppinaShilov, NikolayGinis, PieterShim, Duk-SunGionata, SalviettiShim, JiwookGiordan, DanieleShim, Yoon-Bo Giorgetti, AlessioShimada, KunioGiorgetti, AndreaShin, Dong JinGiorgio, DianaShin, SeokjooGiorgio, IvanShin, Seung WookGiorgio, QuerShinde, SudhirkumarGiorgio-Serchi, FrancescoShintani, YukihiroGiosan, IonShirazi, Mohammad ShokrolahGiovanelli, DavideShirazi, SiamackGiraldo, Juan PabloShojafar, MohammadGirard, SylvainShokrolah Shirazi, Mohammad Girau, RobertoShort, MichaelGiraud, FrédéricShpacovitch, VictoriaGirelli, Valentina AlenaShrestha, Lok KumarGiri, ParitoshShrestha, SudhirGiroud, FabienShrivastava, AatmeshGirousi, StellaShtepliuk, IvanGissane, ConorShu, LeiGiubileo, GianfrancoShukla, Sudheesh K.Giudice, Nicholas A.Shyrokau, BarysGiungato, PasqualeSiadat, MaryamGizzi, LeonardoSiciliani, MarioGjevestad, Jon GlennSidike, PahedingGjoreski, HristijanSidorenko, AlexanderGlas, Dylan F.Sieben, ChristianGlaser, StevenSieberth, TillGlisic, BrankoSiegert, CourtneyGlover, IanSiemes, ChristianGlowacz, AdamSieradzki, RafałGnade, Bruce E.Sierra, BasilioGockenbach, ErnstSierra-Rosales, PaulinaGodina, RaduSiever, BillGodinez Tello, RichardSigaud, OlivierGoecke, Sven-FSignoroni, AlbertoGoers, LisaSiirtola, PekkaGoethals, PauwelSijs, JorisGoffredo, MichelaSik-Lányi, CecíliaGoga, NicolaeSikora, MarekGoh, Shu TingSikorski, WojciechGoldoni, EmanueleSil, DevikaGoldsmith, JeffSillanpää, MikaGolnabi, Amir H.Silpasuwanchai, ChaklamGolob, MarjanSilva, Agnelo R.Golub, EyalSilva, CatarinaGómez Mancilla, Julio CésarSilva, Cristiano M.Gomez, EliotSilva, LígiaGomez, Jose M.Silva, Rone Ilídio DaGómez, LuisSilva-Martinez, JoseGomez-Casco, DavidSilverberg, LarryGómez-Cuba, FelipeSim, SunghanGómez-de-Gabriel, Jesús M.Sima, ChaotanGómez-Rodríguez, AlmaSimanjuntak, Firman MangasaGomis-Bresco, JordiSimeonov, Vasil D.Gonçalves, GilSimic, MilanGonçalves, HernâniSimms, Daniel M.Goncalves, Luis MoreiraSimoncelli, SabrinaGonçalves, Luiz M. G.Simonneau, ThierryGoncalves, OlivierSimons, ThomasGonçalves, Paulo Jorge SequeiraSimos, PanagiotisGonçalves, RicardoSing, Swee LeongGong, JieSingamneni, SaratGong, NeilSingh, Ajay VikramGong, PeijunSingh, Atul KumarGong, WeiSingh, DhananjayGong, YongkangSingh, KeshavGonzález Cortés, AraceliSingh, NavragGonzález Peytaví, GracielaSingh, RajbirGonzalez, AlbertoSingh, RanjanGonzález, AlejandroSingh, Surya P.Gonzalez, JoaquinSingh, YogeshGonzalez, JoseSinghal, KunalGonzález, JuliánSingla, MithunGonzalez, Luis SanchezSinha, JasmineGonzalez, MiguelSipal, VitGonzalez, Nelson MimuraŠipoš, MartinGonzalez, RodrigoŠípová, HanaGonzalez, RubenSips, PatrickGonzalez-Aguilera, DiegoSiqueira, AdrianoGonzález-de-la-Rosa, Juan-JoséSiraj, NoureenGonzalez-Ortega, DavidSirjani, MarjanGonzález-Parada, EvaSirmaçek, BerilGonzález-Peña, Rolando J.Sithole, GeorgeGonzález-Romero, ElisaSitnik, RobertGonzalez-Valdes, BorjaSivashankar, ShilpaGonzalvez, Pablo RodriguezSkelin, Ana KuzmanićGoodall, Noah J.Skipwith, Christopher G.Goodarzi, Farhad A.Skjelvareid, Martin HansenGoodin, DouglasSkoglund, MartinGoossen, KeithSkrunes, StineGopal, SucharitaSkrzypczyński, PiotrGopalai, Alpha AgapeSkulimowski, PiotrGope, ProsantaSlaski, GrzegorzGorazd, ŠtumbergerSlavič, JankoGorcin, AliSleewaegen, Jean-MarieGordillo, Jose ManuelSloan, RobinGorodetsky, VladimirSlobodian, PetrGorricho, Juan-LuisSlota, George M.Górski, MarcinSlouka, ChristophGostar, Amirali KhodadadianSmall, AlexGoswami, SantonuSmeu, EmilGotsis, KonstantinosSmiatacz, MaciejGötzelmann, TimoŠmíd, RadislavGouton, PierreSmietana, MateuszGovind Rao, VishalSmilkstein, TinaGovindan, PramodSmirnov, AlexanderGow, JasonSmith, AlisterGowers, SallySmith, BrandonGoyal, RakaSmith, IanGozdowski, DariuszSmith, Jeremy S.Gracia-Espino, EduardoSmith, JimGrafton, MilesSmith, Patrick JGragnaniello, DiegoSmith, RichardGraham, PaulSmith, Stuart T.Grammalidis, NikosSmith, ZacharyGrammatikopoulos, LazarosSmolka, BogdanGranata, FrancescoSmolka, JacekGranda, Juan CarlosSmolyaninov, IgorGranitzer, PetraSmulko, JanuszGrassani, DavideSnellen, MirjamGrasso, Marco LuigiSo, HongyunGrasso, MarzioSo, JungminGrattieri, MatteoSoar, JeffreyGravenkamp, HaukeSoares, Vasco N.G.J.Graves, Sarah J.Sobolev, Nikolai AndreevitchGravina, RaffaeleSobral, AndrewsGrazia, Carlo AugustoSobrino, Xosé Antón VilaGraziani, SalvatoreSobron, IkerGraziano, Maria DanielaSocorro, Abián BentorGreco, LucaSoh, Jun HuiGreen, DavidSohel, FerdousGreidanus, HarmSohgawa, MasayukiGreminger, MichaelSohn, Hong-GyooGrenzdörffer, GörresSöken, Halil ErsinGrewal, YadveerSolanas, AgustiGriep, MarkSolari, FabioGriffin, ChristopherSoleimani, ManuchehrGriffiths, PeterSoler Aznar, MariaGriggio, FlavioSoleymani, LeylaGrigorie, TeodorSolhusvik, JohannesGrill, RomanŠolić, PetarGrimaldi, DomenicoSoliman, Abdel-HamidGrimaldo, EduardoSoliman, HamdyGrobnic, DanSoliman, MohamedGrochla, KrzysztofSolimene, RaffaeleGroen, Thomas A.Solin, ArnoGroenli, Tor MortenSolis Alfaro, JorgeGrolinger, KatarinaSolmaz, GuerkanGrosch, TheodoreSomà, AurelioGross, Jason N.Son, DongheeGrossi, MarcoSon, JunggabGroves, Roger M.Son, YunsikGruber, JonasSonato, AgneseGruev, ViktorSong, AiguoGruić, IgorSong, ChengGrulkowski, IreneuszSong, EdwardGrussenmeyer, PierreSong, GangbingGu, BoSong, HoubingGu, ChengSong, JibinGu, HaichangSong, KaichenGu, HuanSong, MinkyuGu, KaiSong, QingyangGu, WentingSong, RafaelGU, YuandongSong, ShuangGu, YujieSong, Woo JinGualandi, AdrianoSong, ZhanGualda, DavidSong, ZhiguangGuan, HaiyanSonobe, ReiGuan, YanSood, RohanGuan, YongSophocleous, MariosGuan, YuSorge, ChristophGuarnera, Giuseppe ClaudioSoriano, Julio GómezGüemes, AlfredoSoroushmehr, RezaGuermandi, MarcoSośnica, KrzysztofGuerra, RuiSotelo, RafaelGuerrier, StéphaneSoto, RobertGuerrieri, AntonioSotomayor, MariaGuerriero, FrancescaSoulère, LaurentGui, guanSoundappan, ThiagarajanGui, ZhipengSousa Pavani, GustavoGuidi, FrancescoSousa, Armando JorgeGuidi, VincenzoSousa, FernandoGuido, GiuseppeSousa, HelderGuiotto, AnnamariaSouza, FranciscoGuitton, AlexandreSouza, Paulo DeGuivant, JoseSpaccini, DanieleGuler, UrcanSpachos, PetrosGulrez, Tauseef Spaggiari, AndreaGuna, JožeSpagnolo, VincenzoGunawidjaja, RaySpanakis, Emmanouil GGüntner, AndreasSparacino, GiovanniGuntuku, Sharath ChandraSparkes, ValerieGuntupalli, LakshmikanthSparks, DarrellGuo, BinSpeck, Andrew J.Guo, ChuanfeiSpegazzini, NicolasGuo, FeiSpinazzè, AndreaGuo, FuchunSpinsante, SusannaGuo, LixinSpivak, ArthurGuo, MeifengSpivak, ArturGuo, MengSpizzichino, ValeriaGuo, QingboŠprager, SebastijanGuo, ShuangZhuangSpratford, WayneGuo, WeiSpringer, ShmuelGuo, YaoSquicciarini, GiacomoGuo, YuzhuSrinivas, UmamaheshGurbuz, Ali CaferSrinivasan, MandyamGurbuz, Sevgi Z.Srinivasan, SeshadhriGÜRCAN, ÖnderSrinivasan, SudharsananGurov, IgorSriram, RashmiGut, Jorge Andrey WilhelmsSt. Luce, MervinGuterman, HugoStadlober, BarbaraGutiérrez, DaniaStafford, RickGutiérrez, JonStaglianò, DanieleGutiérrez, Juan ManuelStampach, RadimGutiérrez-Capitán, ManuelStancic, IvoGuvenc, IsmailStančin, SaraGuzek, DominikaStanic, SamoGuzman-Sepulveda, Jose RafaelStanković, Miloš S.Gwak, JeonghwanStantchev, VladimirGyllencreutz, RichardStanwix, PaulGyrard, AmelieStaruch, MargoHa Lee, ByeongStassen, IvoHa, MinkeunStateczny, Andrzej EugeniuszHaack, BarryStathis, Stiros C.Haas, AndreasStatkiewicz-Barabach, GabrielaHaas, RüdigerStatsyuk, Natalia V.Häberlin, AndreasStaubli, ThomasHabisreuther, TobiasStavropoulos, PanagiotisHadas, TomaszStavropoulos, Thanos G.Haddag, BadisStavroulakis, GeorgiosHadjimitsis, DiofantosStefanakis, NikolaosHaga, NozomiStefanescu, AlexandraHaggag, SherifStefani, AlessioHagler, Gayle S.W.Stefania, MonicaHahn, MichaelStefaniak, PawełHahn, UlrichStefanov, AndrejHaick, HossamStefanov, KonstantinHaidar, AhmadSteffen, HolgerHaitjema, H.Steinberg, Steven J.Hajder, LeventeSteinhauer, StephanHájek, PetrSteinmetz, Nicholas A.Hajiahmadi, M. RezaStępień, GrzegorzHajjar, Jerome F.Stępień, KrzysztofHajný, JanStepinski, TadeuszHalder, ArnabŠter, BrankoHalkon, BenStergiou, DimitrisHalldórsson, SkarphéðinnSteri, GaryHalliday, DavidSterling, DavidHalmen, CekiSternberg, Ben K.Hamadache, MoussaStetter, RalfHamad-Schifferli, KimberlyStevens, EricHamann, EliasStevens-Navarro, EnriqueHamari, JuhoStewart, Linda D.Hamid, MohamedStewart, SarahHamida, HallilSteyn, WillemHamilton-Fletcher, GilesSTHAL, FabriceHammer, Nathan I.Stiens, JohanHampel, DanielaStifter, DI MatthiasHan, BaoguoStins, JohnHan, ChangseokStirling, LeiaHan, Feng-TianStocker, MarkusHan, JinluStoffelen, AdHan, JungheeStokic, DraganHan, JungongStoliar, PabloHan, KijunStoll, EnricoHan, MingStolz, RonnyHan, QunStopa, Justin E.Han, TaoStorch, TobiasHan, YoukyungStork, WilhelmHan, ZandongStory, BrettHancock, Craig MatthewStrakowski, MarcinHane, KazuhiroStrano, MichaelHanke, KlausStrauss, AlfredHansberger, Jeffrey T.Strehlitz, BeateHänsch, RonnyStrelcov, EvgheniHans-Joachim, KrauseStrelniker, Yakov M.Hao, ChengpengStrle, DragoHao, PengStrle, GregorHappee, RienderStrohm, EricHar, DongsooStrollo, Antonio G.M.Hara, ShinsukeStronks, H ChristiaanHardwick, Stephen RStruk, PrzemyslawHardy, John G.Strumiłło, PawełHarezlak, KatarzynaStruzik, MichalHarish, SivasankaranStrzelecki, MichalHärkönen, JaakkoStuckenschmidt, HeinerHarnois, MaximeSTUERGA, DidierHarris, PaulŠtumberger, GorazdHarrison, WillieStumpf, AndreHartbauer, ManfredStumpf, Richard P.Hartmann, MarkusSturtevant, BlakeHartmann, Mitra J. Z.Stylianidis, EfstratiosHashemi, EhsanSu, BinHashemi, MatinSu, DaobiligeHashemi, MortezaSu, HongboHashimoto, HiroshiSu, JinyaHasni, HasseneSu, JudithHassan, ModarSu, Ming-ShouHassan, Mohammad MehediSu, Pi-GueyHassan, MuhammadSu, QinglinHata, AlbertoSu, RuoyuHatfield, JerrySu, Shun-FengHaug, Anton J.Su, Tung-ChingHautefeuille, MathieuSu, YenningHavlík, JanSu, YishanHawes, MatthewSu, Yi-ShengHayajneh, Khaled F.Su, Yuan-FongHayajneh, ThaierSua, Yong MengHayashi, HisashiSuarez, AlvaroHayashi, TakahiroSubasi, AbdulhamitHayes, PatrickSubbaraman, LakshmanHe, ChengbingSuciu, GeorgeHe, DebiaoSuematsu, KoichiHe, JasonSugano, MasashiHe, JinSugawa, KosukeHe, Jing SelenaSugimura, DaisukeHe, JinxinSuh, Doug YoungHe, LimingSuh, MinahHe, RuiliSuh, Myoung-GyunHe, ShiboSuh, Young SooHe, SuiningSuhr, Jae KyuHe, TianSui, YaoHe, YijunSukhov, SergeyHeberger, KarolySullivan, AndrewHebrard, LucSullivan, CarlHedayati, MehdiSullivan, Pierre E.Hedegaard, BrockSullivan, Rani W.Hedley, MarkSulzer, PhilippHedrick, Tyson L.Sumetsky, Michael (Misha)Hegyi, AlexSummerscales, JohnHeidari, HadiSun, Daniel (Jian)Heien, MichaelSun, GuangcaiHeilbronner, UrsSun, GuanghaoHeise, RainerSun, GuodongHeiselberg, HenningSun, HaoHeiskanen, ArtoSun, HongyuHejazin, YazanSun, JieHelfricht, KaySun, JiliHellbrück, HorstSun, JinpingHeller, Maija IrisSun, KangHemm-Ode, SimoneSun, Kien WenHenesey, LawrenceSun, MengHeng, LionelSun, NianHenning, BerndSun, NingHeo, JinseokSun, QingsongHer, Shiuh-ChuanSun, ShaohuiHeredia-Guerrero, José AlejandroSun, ShuliHerkommer, AloisSun, WeizhengHermoso, RamónSun, XinghuaHernaez, MiguelSun, XiulanHernández Callejo, LuisSun, YanguoHernández Sosa, José DanielSUN, YanhuaHernández, ÁngelaSun, YongmeiHernández, David NaranjoSun, YongxiaHernández, Margarita GonzálezSun, Yung-ShinHernández, VicenteSun, YuxiangHernandez, WilmarSun, ZhanliHernandez-Lopez, DavidSun, ZhenglongHernández-Pajares, M.Sun, ZhiliHernández-Ramos, José L.Sung, Chang KyungHerout, AdamSung, KiWonHerrera, Edith PulidoSun-Kyu, LeeHerrera, LuisSunny, S. M. Nahian AlHerrera-May, AgustinSuntharalingam, KogularamananHerrero, Jesus GarciaSurmacki, JakubHerrero-Agustín, José-LuisSusilo, EkawahyuHerrero-Tejedor, Tomás RamónSutin, AlexanderHerry, Christophe L.Suzuki, KenichiroHersek, SinanSuzuki, MasakiyoHervás Lucas, RamónSuzuki, ReijiHerzog, Joseph B.Suzuki, TaroHespel, LaurentŠvanda, MilanHesselbarth, AnjaSvedendahl, MikaelHeyd, RodolpheSvendsen, Morten Bo SøndergaardHianik, TiborSvorc, LubomirHicke, KonstantinSwami, NathanHigurashi, EijiSwartz, R. AndrewHikage, TakashiSweetman, MartinHilfer, MichaelSwiderski, WaldemarHill, David J.Synnott, JonathanHill, RichardSyrový, TomášHill, SamuelSyrris, VasileiosHillel, Aharon BarSysoev, VictorHiller, KarlaSzabo, AlexandreHiller, SusanSzabo, LorandHilleringmann, UlrichSzczepanik, BeataHillman, A.R.Szczęsna, AgnieszkaHinderliter, BrianSzekely, GyorgyHinostroza, IsraelSzeląg, BartoszHippler, MichaelSzeląg, WojciechHirdaris, SpyrosSzénási, SándorHiromori, AkihitoSzeto, Grace P. Y.Hirtle, Stephen C.Szewczyk, RomanHo, DanielSzkaliczki, TiborHo, MichaelSzöllősi, DanielHo, Siu ChunSzulwic, JakubHo, Siu WaiSzwoch, MariuszHo, Tsung-YiTabatabaeenejad, AlirezaHobbs, StephenTabib-Azar, MassoodHoblos, GhalebTaetz, BertramHodgkinson, JaneTafti, AhmadHoffman, Matthew J.Taghvaeian, SalehHoffmann, KlausTaguchi, Y-h.Hoffmeister, DirkTahara, HironobuHojo, Masaru K.Taheri, ParsaHolade, YaoviTajadura, AnaHölbl, MarkoTajbakhsh, Shahriar EtemadiHolder, LawrenceTak, SehyunHolder, Simon J.Takami, AkinoriHoller, StephenTakashima, KazutoHolmes, ChristopherTakebayashi, HidekiHolobar, AlešTakeda, RyoHolst, ChristophTakemura, HiroshiHolz, ChristianTakizawa, HotakaHolzinger, AndreasTaklo, Maaike M VisserHolzinger, MichaelTalaga, PaulHomayouni, saeidTalakoub, OmidHondori, HosseinTalla, VamsiHonegger, DavidTalleb, HakeimHoneine, PaulTallman, Tyler N.Hong, Chang-KiTalukdar, TanveerHong, Keum-ShikTalvitie, JukkaHong, MenTam, Gary K.L.Hong, QingyangTAMARIN, OllivierHong, SungjunTamer, AykutHong, WenjingTamil, LakshmanHong, WonbinTamošiūnas, VincasHong, Youn-SikTamura, HirokiHong, YuningTan, BoHonkavaara, EijaTan, Do DuyHontañón, EstherTAN, JiajieHooley, Emma NicoleTan, JingHopmann, ChristianTan, KunHopp, TorstenTan, LianshengHoran, BenTan, SayHorauer, MartinTan, ShurunHoreman, TimTan, Yew TeckHorita, FlávioTanabe, TadaoHorng, Shi-jinnTanaka, MasayukiHorng, Tzyy-ShengTananaiko, OksanaHorri, NadjimTang, FujianHorrillo Güemes, CarmenTang, HaoHorrillo, María Del CarmenTang, LihuaHorsfall, Alton B.Tang, Lu-AnHortal Quesada, EnriqueTang, Tiffany YaHorvat, MarkoTang, WenwuHorvath, GyörgyTang, XinhuaHorvath, RobertTang, YiHoshyarmanesh, HamidrezaTang, ZhichuanHossain, A.K.M. MahtabTanganelli, GiacomoHossain, AlamgirTani, JacopoHossain, AzadTanil, CagatayHossain, Md ShafaeatTao, KaiHossain, ShamimTao, LeiHosseini, NiloufarTao, LiHou, ShuangTapetado, AlbertoHou, YiTapete, DeodatoHouben, SebastianTAPU, RuxandraHoussineau, JeremieTarabini, MarcoHoward, DavidTarantino, CristinaHowarth, Samuel J.Tardós Solano, Juan DomingoHoxley, DavidTarricone, LucianoHoyhtya, MarkoTartarisco, GennaroHoznedl, MichalTaruno, AkiyukiHrad, JaromirTasoulis, SotirisHrishikeshavan, VikramTassou, ChrysoulaHristoforou, EvangelosTatar, ErdincHsiang, Hsing-ITatar, Mihai OlimpiuHsiao, Fu-yuenTatlas, Nicolas-AlexanderHsiao, Rong-ShueTaulu, SamuHsiao, Shen-FuTauro, FlaviaHsiao, Ta-ChihTavakolian, KouhyarHsiao, Yung-ChiaTavassolian, NegarHsieh, Chia-LungTawfik, Aly M.Hsieh, Fu-ShiungTaylor, JoeHsu, Heng-TungTaylor, NathanielHsu, Huan-HsuanTedetti, MarcHsu, Jin-ChenTee, Benjamin C.K.Hsu, Jui-TingTeh, Kah ChanHsu, Kuo-WeiTeixeira, César A. D.Hsu, Ming-weiTeixeira, FernandoHsu, Ting-YuTeixeira, Fernando A.Hsu, WensyangTeixeira, Marcos F.S.Hsu, Yu-LiangTeixido, TeresaHu, DanyingTelling, Jennifer W.Hu, F.Temko, AndriyHu, Fang Q.Tennina, StefanoHu, FeiTeo, Tee-AnnHu, LiangTeodorescu, FlorinaHu, MiaoTeodoro, Ana CláudiaHu, NingTeranishi, NobukazuHu, RuiTerebes, RomulusHu, ShaohanTeresa Sánchez, MaríaHu, WeiTerra, Fabricio Da SilvaHu, WeiduoTerroso-Saenz, FernandoHu, XiaogongTervonen, Jouni K.Hu, Yu HenTerzić, KasimHu, ZhongxuTesei, AlessandraHuang, Bo WunTestoni, NicolaHuang, ChangTeti, RobertoHuang, Cheng-LiangTetreau, GuillaumeHuang, Chih-ChingTeunissen, PeterHuang, Chih-WeiTeymourian, HazhirHuang, Chih-YuanThalman, RyanHuang, Chin-YaThanh, Kien NguyenHuang, ChongTheel, OliverHuang, Chung-ChiTheiler, JamesHuang, HaoshengTheodorakopoulos, IliasHuang, Hsin-HaouTheodoulidis, TheodorosHuang, JennyTheuwissen, AlbertHuang, JiangshuaiThiebes, BenniHuang, JianjunThiel, MichaelHuang, JiaruiThielemann, ChristianeHuang, JieThielens, ArnoHuang, JingfengThoeni, KlausHuang, LipingThomas, Jean BaptisteHuang, PanfengThomas, SanjuHuang, Po-TsangThomas, Varghese AntonyHuang, QijinThomson, NeilHuang, QinghuaTian, DaxinHuang, QinlongTian, GuiyunHuang, Shih-ChangTian, JiaojiaoHuang, Shih-ChiaTian, JieHuang, WeiminTian, YingtaoHuang, WenliTian, ZengshanHuang, XianTian, ZhenhuaHuang, XiaodongTiemann, JanisHuang, XinboTimopheev, AndreyHuang, YanboTin, ChungHuang, YaoxianTing, Ching-HuaHuang, YingTixier-Mita, AgnesHuang, YishuoTjin, Swee ChuanHuang, Yo-PingTkáč, JánHuang, YuanheToda, MasayaHuang, Yu-ChiehTognetti, AlessandroHuang, Yu-HsiToietta, GabrieleHuang, Zi-GuiTojo, NaokiHuber, MarcoTokuda, TakashiHubert, FrédéricToledano-Ayala, ManuelHubin, AnnickToledo, Claudio Fabiano MottaHudoklin, DomenTollner, Ernest W.Huertas, César SánchezToma, Daniel MihaiHufert, Frank T.Toma, KojiHugel, VincentTomaiuolo, GiovannaHughes, DanaTomarchio, OrazioHughes, IfanTomasi, MatteoHughes, RobertTomassetti, MauroHuh, Hong-WookTombelli, SaraHuh, Yun SukTombesi, SergioHui, GuoHuaTomida, Akemi GalvezHuisman, GijsTomitaka, AsahiHultmark, MarcusTomko, MartinHung, Ho-LungTon, Shan-ShinHunger, DavidTong, LiangHunt, AndrewTong, ShengHunze, ArvidTong, YanhongHuo, XingliangToniolo, RosannaHuotari, MattiTõnisson, HannesHusak, Gregory J.Tonoli, AndreaHussain, MunawarTopolyan, IrynaHuston, DryverTopouzelis, KonstantinosHutchins, DavidTordera, Eva MarínHutter, TanyaTöreyin, HakanHutu, Florin DoruTorres, Pedro M. B.Huynh, Tan-PhatTorres, SergioHwang, Chi-HungTorres-Gomez, IsmaelHwang, DavidTorres-Luque, MagdaHwang, Dong-HwanTorres-Rua, AlfonsoHwang, Han-JeongTorres-Sospedra, JoaquínHwang, Keum CheolTorroba, TomasHwang, WanSikTortolini, CristinaHwu, Jenn-GwoTortschanoff, AndreasHyun, EuginTorunbalci, Mustafa MertHyun, Woo JinToth, CharlesIacomi, FeliciaTothill, IbtisamIbaraki, SoichiTouhafi, AbdellahIbarlucea, BergoiTouya, GuillaumeIbarra-Castanedo, ClementeTragos, Elias Z.Iborra, EnriqueTrainiti, GiuseppeIbragimov, BulatTramarin, FedericoIckowicz, AdrienTrammell, Scott A.Ide, ReikoTran, Binh Q.Idrees, HaroonTran, Canh-DungIervolino, PasqualeTran, Gia KhanhIglesias Martínez, José AntonioTran, Huy Iglesias Rodríguez, RobertoTran, Le-NamIglesias, Carlos BorregoTran, Quoc-HuyIhantola, SakariTran-Quang, VinhIkeda, AkihikoTrashin, StanislavIkeno, ShinyaTraver, VicenteIkki, SalamaTravieso-Gonzalez, Carlos M.Ilarri, SergioTrebar, MiraIllie, AdrianTreloar Padovano, HayleyIlmonen, PauliinaTremeau, AlainIm, HyungsoonTrevathan, JarrodImani, Mohammadreza F.Trigona, CarloImperatore, PasqualeTrigo-Rodríguez, Josep M.Imran, MohammadTrilles, SergioImran, MuhammadTrimmel, MichaelInan, Omer T.Trinder, John C.Inci, FatihTristant, DamienIndri, MarinaTrivero, PaoloIngebrandt, SvenTriviño, AliciaInnocent, ManuelTrobec, RomanIno, KosukeTroiani, EnricoIoakimidis, Christos S.Tron, RobertoIoannidis, DimosthenisTroncoso-Pastoriza, Juan RamónIoannis, KonidakisTrono, CosimoIonescu, AnisoaraTrontelj, JanezIosa, MarcoTrouillon, RaphaëlIova, OanaTrovati, MarcelloIqbal, RahatTrowell, StephenIrannejad, MehrdadTroynikov, OlgaIsaad, JalalTrung, Ngo ThanhIsada, TomonoriTrung, Tran QuangIsalgue, AntonioTruong-Hong, LinhIshaq, IsamTsai, FuanIshigami, GenyaTsai, JichiangIshikawa, MasatoTsai, Men-ShenIshizaki, KenjiTsai, MichingIshmanov, FarruhTsai, Ming-JongIslam, S.M. RiazulTsai, Pei-WeiIslam, ShareefulTsai, Wan-YuIslam, SyedTsai, Wen-ChiIssac, BijuTsai, Yao-WenIto, DaisukeTsai, Yi-Chang (James)Ito, SoTsakalof, AndreasIto, TsukasaTsaloglou, Maria-NefeliItoh, ToshihiroTsang, Kim FungItoh, ToshioTsangouri, EleniItoh, YutaTsao, Hen-WaiIulia, Gabriela DavidTsaousis, Konstantinos TIvakov, AlexanderTse, Ming-Leung VincentIvǎnescu, Nick AndreiTseles, DimitriosIvanov, AleksandarTseng, Chao-HsiungIvanović, MirjanaTseng, Fan-HsunIverson, Nicole M.Tseng, KevinIwasaki, AkiraTseng, Po-HsuanIwuoha, Emmanuel I.Tseng, Wei-LungIyota, TaketoshiTsigaridas, GeorgiosIzu, NoriyaTsigaridas, Georgios N. Izumi, ShintaroTsiotras, PanagiotisJabari, ElaheTsioukas, VassiliosJabari, ShabnamTsipouras, MarkosJachowicz, RyszardTsiropoulou, Eirini EleniJackson, JosephTsoukalas, DimitrisJackson, NathanTsoulos, Ioannis G.Jacobs, PeterTsow, Francis (Tsing)Jacobsen, KarstenTsunenori, MineJacobsen, Knut StanleyTsunoda, NaoyaJacobsen, Rune HylsbergTsyganova, Julia VJadeja, RaviTu, ChengJaeheung, LeeTu, ChuanjunJafari, SoheilTU, EnmeiJafarkhani, RezaTu, JinchunJaffrezic-Renault, NicoleTu, Shan TungJaganathan, ArunTU, WeiJaimes, Luis G.Tucci, GraziaJain, ShubhamTuma, JiriJakiela, SlawomirTung Chong, David WongJakobson, ClaudioTung, Tran ThanhJakubik, Wiesław P.Tuominen, SakariJalal, AhmadTur, MosheJalali, RezaTura, AndreaJalaliniya, ShahramTurchetta, RenatoJames, MichaelTurco, AntonioJamone, LorenzoTurcza, PawelJamrozik, WojciechTuriel, Javier PérezJan, Shau-ShiunTurkanović, MuhamedJang, Jae-WonTurkmen, MustafaJang, JuwookTurksoy, KamuranJang, Kyung-InTurmo, JoseJang, TaekwangTurner, ChrisJang, YeongTurner, DarrenJang, YoungkyoonTurovets, SergeiJaniesch, ChristianTuset-Peiro, PereJanik, PawełTutsch, RainerJanissen, RichardTuttlies, UteJankowski-Mihułowicz, PiotrTuukkanen, SampoJanner, DavideTylecek, RadimJanošek, MichalTzallas, AlexandrosJansen, MarcusUbertini, FilippoJansen, Rob G.Uche, ChibuezeJanssen, KrisUchiyama, HideakiJansson, Jussi-PekkaUd Din, AmadJara, Carlos A.Udalcovs, AleksejsJarchi, DelaramUdd, EricJaros, JiriUddin Ahmed, MobyenJaroszewicz, Leszek R.Udovydchenkov, IlyaJärvinen, KimmoUeda, TaroJasinski, GrzegorzUeno, TetsuroJaskolka, JasonUgbolue, UkadikeJáuregui-Vázquez, DanielUhring, WilfriedJau-Sheng, WangUjihara, MasakiJavaid, Ahmad Y.Ulapane, NalikaJavanmard, MehdiUlman, VladimírJaw, Jen-JerUme, I. CharlesJayamohan, HarikrishnanUmeda, HirokiJayaraman, ChandrasekaranUmek, AntonJayaraman, PremUngurean, IoanJe, ChangsooUnland, RainerJędrzejewska-Szczerska, MałgorzataUnnikrishnakurup, SreedharJedrzejewski, Kazimierz PiotrUno, ShigeyasuJeng, YihUnzueta, LuisJenkins, KennethUr Rehman, MasoodJenkins, R. BrianUrasinska-Wojcik, BarbaraJensen, RyanUrbanek, JacekJeon, Kwang MyungUrbanelli, LorenaJeon, MansikUrbieta, AitorJeon, SanghunUria, NaroaJeon, SangminUriarte, Rafael BrundoJeon, Sang-WoonUrniezius, RenaldasJeong, Hoon EuiUrricelqui, JavierJeong, Nak CheonUrwyler, PrabithaJeppesen, JesperUsamentiaga, RubénJesenik, MarkoUsländer, ThomasJesús García Domínguez, JuanUslar, MathiasJesus, Sérgio M.Uto, KuniakiJha, Devesh K.Uysal, FarukJhumka, ArshadUzel, SebastienJi, HaoUzkent, BurakJi, HongliUzunoğlu, AytekinJi, KefengVacavant, AntoineJi, ZhanlinVacher, MichelJia, ShaofengVaculovičová, MarketaJia, YuVagapov, YuriyJia, YunjianVagedes, JanJia, ZhenzhongVai, Mang I.Jiang, ChunxiaoVaisocherová - Lísalová, HanaJiang, DaiVakhitov, AlexanderJiang, DanchiValdez, FevrierJiang, Dong XiangValdivia Y Alvarado, PabloJiang, HongboValença, JonatasJiang, RuinianValencia, José F.Jiang, XinValencia, RafaelJiang, XuchuanValente, AntonioJiang, XunfeiValente, VirgilioJianga, ShangValentini, GabrieleJimenez, CristoferValentini, Pier PaoloJimenez, FelipeValenzuela, JuanJimenez, LuisValera, EnriqueJimenez, RicardoValerio, GuidoJiménez-Herranz, José MiguelValiente, DavidJiménez-Martínez, RicardoVallabhajosula, SrikantJimeno-Morenilla, AntonioValladares, CesarJin, HongxiaoVallan, AlbertoJin, JiongVallati, MauroJin, MaolinVallejos, StellaJin, TianValova, IrenJin, XiVan Bennekom, W.P.Jin, XiaohangVan Caenegem, OlivierJin, XiuliangVan Camp, MichelJin, Xue-BoVan Den Bossche, MichaelJin, YierVan Den Brom, H.E.Jin, ZhigangVan Der Vegte, W.F.JING, JinVan Der Zande, DimitryJing, QingshenVan Dinh, AnhJiří, ŠebestaVan Do, TienJiri, SilhaVan Gemert, JanJo, HongkiVan Genderen, PietJoerger, MathieuVan Gerven, P.W.M.Johannes, Matthew S.Van Heerden, KirillJohansson, MalinVan Hees, VincentJohn, DineshVan Helleputte, NickJohn, SamVan Hoof, ChrisJohnson, Blake N.Van Huffel, SabineJohnson, BrianVan Opstal, JonnaJohnson, ColinVan Roosbroeck, KatrienJokinen, OlliVan Schooten, Kimberley S.Jolliffe, KatrinaVan Someren, MaartenJolly, PawanVan Stan, John T.Jones, SteveVan Werkhoven, HermanJones, Trevor G.Van Winden, StevenJones, W LinwoodVan Wittenberghe, ShariJong, Gwo-jiaVanegas Alvarez, FernandoJoo, Sang-WooVanek, BalintJoordens, MatthewVangelista, LorenzoJosé García-Peñalvo, FranciscoVanli, O.ArdaJosé Ramiro, Martínez De DiosVanrumste, BartJosé, Pazos AriasVanus, JanJoshi, NiravkumarVardanega, TullioJouandeau, NicolasVardasca, RicardoJouaneh, MusaVardoulis, OrestisJoung, Hyou-ArmVarga, MariánJoutsijoki, HenryVargas-Rodriguez, EverardoJozwik, MichalVargas-Rosales, CésarJu, HeongkyuVargis, ElizabethJu, ZhaojieVarón, MargaritaJuang, Jyh-ChingVarriale, AntonioJulea, AndreeaVasconcelos, FranciscoJun, ImaokaVashist, Sandeep KumarJung, AndrasVasilakos, ChristosJung, Eun SungVasilateanu, AndreiJung, HaejoonVasilescu, AlinaJung, Ho-SupVasques, FranciscoJung, JaehanVasseur, PascalJung, JiyoungVassiliadis, SavvasJung, MerelVasylieva, NataliaJung, MinchaeVatavu, AndreiJung, YunhoVathy-Fogarassy, ÁgnesJuodkazis, SauliusVaughan, NeilJuránek, RomanVauzeilles, BorisJurdak, RajaVázquez Bermúdez, DavidJuskowiak, BernardVazquez Besteiro, LucasJussila, JariVázquez Corral, JavierKaabouch, NaimaVazquez Garcia, CarmenKaajakari, VilleVázquez, Carlos Andres LunaKabir, K. M. MohibulVazquez, MaribelKabiri Ameri, ShidehVčelák, JanKacem, NajibVedady Moghadam, Mohammad RezaKacik, DanielVelastin, Sergio AKaczmarek, MariuszVelázquez, RamiroKaczmarek, MichałVelez, GorkaKadimisetty, KarteekVelisavljevic, VladanKaewunruen, SakdiratVella, FilippoKafle, SwatantraVelotta, RaffaeleKagawa, KeiichiroVeluvolu, Kalyana C.Kahirdeh, AliVenckauskas, AlgimantasKaimakamis, Evangelos K.Venckevičius, RimvydasKaipa, Krishnanand N.Venditti, IoleKaiser, AndreasVendittozzi, CristianKaiwartya, OmprakashVenkatakrishnan, AnushaKalamkar, Sanket S.Venkatesan, RamachandranKalantar-Zadeh, KouroshVentura, Joao OliveiraKalayci, Tahir EmreVera, EstebanKalayci, TunaVerboven, ErikKaleita, AmyVerboven, PieterKalisky, BeenaVerbovšek, TimotejKalivas, GrigoriosVerbree, EdwardKaliviotis, StathisVerde, FrancescoKalkman, JeroenVerde, SimonaKalogiros, JohnVerdini, FedericaKaltsas, GrigorisVerdoliva, LuisaKalwat, Michael AVergari, ClaudioKameda, MasaharuVerhaevert, JoKamsu-Foguem, BernardVerikoukis, ChristosKan, TetsuoVerma, AmitKandpal, Lalit MohanVerma, RoliKandris, DionisisVeronese, FabioKaneta, TakashiVerstraeten, WillemKang, DaeshikVertruyen, BenedicteKang, Do HyunVervisch, BramKang, Dong-KuVerwaeren, JanKang, DongyangVespignani, MassimoKang, Hang-BongVetrov, Stepan YaKang, HyunchulVettore, AntonioKang, InpilVezzetti, EnricoKang, Je-WonViani, FedericoKang, KaiVicentini, FedericoKang, Li-WeiVidal-Verdu, FernandoKang, Moon GiVidic, JasminaKang, NingVidoni, RenatoKang, Soon JuViegas, DianaKang, Suk-JuVieira, DarioKang, TaeghyunVieira, VaninhaKangas, MaaritVien, Quoc-TuanKaniewski, PiotrViik, JariKanik, MehmetVikas, VisheshKanoun, OlfaVilajosana, XaviKantarci, BurakVilanova, XavierKanter, Theo G.Vilela, DianaKantola, RaimoVilella, EvaKanyong, ProsperVilla, FedericaKaplan, AndreyVilla, PaoloKaplon, JanVillar, JoseKapuscinski, TomaszVillarroel, Grover ZuritaKarabchevsky, AlinaVilleneuve, EmmaKaragianni, EvangeliaVincze, MarkusKaratzas, KostasVinogradov, SergeyKarcher, DarrinVinogradov, Sergey L.Karczmarek, PawełViollet, StéphaneKari, ChadiViprey, FabienKarim, Karim S.Virdis, AntonioKarim, Mohammad RezaulVirkki, JohannaKarim, RashedVirmontois, CédricKarimi, HamidVirtanen, SeppoKarimi, HassanVirzonis, DariusKarimova, SvetlanaVishwanath, KarthikKarmakar, NemaiVítek, StanislavKarnaushenko, DaniilVitelli, MassimoKarnowski, KarolViter, RomanKaroń, GrzegorzVitetta, GiorgioKarp, MattiVittuari, LucaKartsakli, ElliVitucci, Enrico MariaKarus, AvoVivanco, Alfonso PadillaKaryotis, VasileiosVlachokostas, AlexKašalynas, IrmantasVo, Ba-NguKashif, AliVoelkel, ReinhardKashino, KunioVogel, ThomasKasneci, EnkelejdaVoglhuber-Brunnmaier, ThomasKasprowski, PawełVohland, MichaelKassab, MohamadVoiculescu, IoanaKasser, MichelVoisin, SophieKastner, WolfgangVoitechovic, EditaKataoka, HirokatsuVollaire, ChristianKatasonov, ArtemVollebregt, StenKateris, DimitriosVolmer, MariusKato, FumitakeVolná, EvaKatsaros, KonstantinosVolosencu, ConstantinKatsikas, SokratisVornicu, IonKatwal, GiwanVoros, Nikolaos S.Kaur, AmandeepVos, StevenKaur, AmardeepVosoughi, AidaKauranne, TuomoVourlitis, George L.Kaushik, AjeetVoutetaki, Maria–StylianiKawabata, KohsukeVouyioukas, Demosthenes D.Kawalec, AdamVozel, BenoitKawamura, KensukeVozzi, GiovanniKaya, TolgaVreugdenhil, MarietteKaynak, AkifVu, Ngoc SonKazimierski, WitoldVukovic, NatashaKazuhiro, KondoVulić, MilivojKažys, Rymantas JonasVulpe, AlexandruKeating, AdrianVuppala, SatyanarayanaKędzierski, MichałVu-Van, HiepKefauver, Shawn C.Vyskočil, VlastimilKehl, FlorianWachsmann-Hogiu, SebastianKeirsbulck, LaurentWackerlig, JudithKeiser, GerdWADA, ChikamuneKelb, ChristianWada, DaichiKeller, AdrianWade, Eric R.Kelly, MatthewWadhwa, ManishKelner, JanWagatsuma, HiroakiKemmegne-Mbouguen, Justin ClaudeWage, KathleenKeogh, EamonnWagner, BernardoKeoh, Sye LoongWagner, Stefan RahrKereszturi, GaborWagner, StevenKerhervé, HugoWagner, ThorstenKerns, JemmaWahaia, FaustinoKersting, UweWahrburg, ArneKeshavarz Hedayati, MehdiWajrak, MagdalenaKeshtkaran, Mohammad RezaWalczak, KrzysztofKeszycka, Maria MarkiewiczWaldvogel, Siegfried R.Ketcheson, ScottWalendziuk, WojciechKeusgen, MichaelWales, Dominic J.Khalaf, WalaaWalker, AlanKhaleghi, MortezaWalker, Ross M.Khalili, AshkanWalker, Stuart D.Khaluf, YaraWalker, Thad GKhan, AmirWallace, R BruceKhan, HamzaWallhoff, FrankKhan, ImranWalsh, KerryKhan, Jamil Y.Walter, Jonathan PKhan, Junaid AhmedWan, FengKhan, KhalidWan, JiafuKhan, MuhammadWan, Kai TaiKhan, Muhidul IslamWan, LiangtianKhan, SaadWan, YiKhan, SaleemWandowski, TomaszKhan, Salman H.Wang, AiminKhan, Salman SaeedWang, AiningKhan, Shehroz S.Wang, BangKhan, WasiqWang, BinbinKhan, ZaheerWang, BinghaoKharkivskiy, SergiyWang, BoxiongKhatibi, Akbar A.Wang, ChengKhatibi, SiamakWang, Chi-KueiKheradmand, AminWang, Chiu-YenKhodabandeh, AmirWang, CongKhodadad, DavoodWang, DongKhodaei, Zahra SharifWang, FangKhodasevych, Iryna E.Wang, FeiKhorov, EvgenyWang, FukeKhosravian, AlirezaWang, Grace YKhwaja, Ahmed ShaharyarWang, GuijinKhyam, Mohammad OmarWang, GuodongKi, Seo JinWang, HaigangKiang, Jean-FuWang, HaiyangKianoush, SanazWang, HaoKibangou, AlainWang, HongquanKibret, BehailuWang, HongyanKiełczyński, PiotrWang, HuaiminKieninger, JochenWang, HuaqingKihara, NaotoWang, HuihuiKijanka, PiotrWang, Hui-MingKikuchi, HiroakiWang, JackKilic, OzlemWang, JianKilinc, DevrimWang, JianfengKim, AyoungWang, JieKim, BeomSeokWang, Jing-MinKim, Byung-SeoWang, JinqiaoKim, Chang HunWang, KanKim, ChangilWang, KevinKim, CheolGiWang, LeiKim, DaeEunWang, LiangminKim, DaewonWang, LidaiKim, Dae-YoungWang, LifengKim, Do YoungWang, LingKim, DonghwanWang, LingLingKim, DonghyunWang, LingyunKim, Du YongWang, LiuchengKim, Eun SokWang, MenghuaKim, EuntaiWang, MengyuKim, Gon-HoWang, MiKim, Hyug-HanWang, Michelle (Yibing)Kim, HyunbumWang, Mu-chunKim, HyungminWang, PengfeiKim, Hyung-SeokWang, Peng-FeiKim, Hyung-SinWang, PengtaoKim, HyunsungWang, PingKim, I. S.Wang, QiKim, Jae ChangWang, QingKim, Jae HongWang, QingQingKim, Jae JoonWang, RanranKim, Ji-HoonWang, RobertKim, Ji-HwanWang, RosalindKim, Jong SeungWang, San-YuanKim, Jong-HoonWang, ShaopengKim, JooheeWang, Sheng-ShihKim, Jung RaeWang, WeiKim, JungsukWang, WenKim, JungyoonWang, Wendy HuiKim, KeonwookWang, WenyiKim, KiilWang, XiaolingKim, KyungkiWang, XiaonaKim, MinKooWang, Xiaopeng Kim, MyunghoiWang, XingweiKim, Robin E.Wang, XinhengKim, Sang JaeWang, XiuminKim, Sang WooWang, YanKim, SangkilWang, YangKim, SeokhyunWang, Yao-tienKim, SeokminWang, YiKim, Seong-EunWang, YideKim, Seung-SepWang, YilongKim, SeyoungWang, YinKim, Soo YounWang, YingminKim, Sung SukWang, YipingKim, Sung-PhilWang, YizhongKim, SunwookWang, YongKim, Tae-HyungWang, YonghuiKim, Tae-seongWang, YongxiangKim, TaesungWang, YuKim, TaewooWang, YueKim, Tai-hoonWang, Yu-LinKim, WonjunWang, YunKim, Yong-HwaWang, YuncaiKim, Young PilWang, Yun-MingKim, Young-DukWang, YunshengKim, YoungokWang, ZengcaiKim, YoungwookWang, ZhaoshengKinet, DamienWang, ZheKing, GregoryWang, ZhengKingma, BorisWang, ZhiguangKiourti, AsiminaWang, ZhipingKirby, RayWang, Zizhong JohnKirchner, Elsa AndreaWann, Chin-DerKirchner, JensWanninger, LambertKirei, Botond SándorWanrooij, M.M. VanKirk, AndrewWanunu, MeniKirkko-Jaakkola, MarttiWan-Wendner, RomanKirkpatrick, Sean J.Ward, JohnKirsanov, DmitryWard, JonathanKisner, AlexandreWard, PhilKist, AlexanderWardlow, BrianKit, DmitryWargocki, PawelKita, NobuyukiWarkiani, Majid EbrahimiKitano, ClaudioWarren, CraigKiviste, MihkelWarren, SeanKleinschmidt, João HenriqueWarren-Smith, StephenKlem, KarelWasicek, ArminKlemm, Richard A.Wasko, ConradKlenk, JochenWatanabe, FumiyaKlepka, AndrzejWatanabe, KenKlucken, JochenWatanabe, ShinichiKlueh, UlrikeWatanabe, TetsuyouKnauth, StefanWatel, DimitriKnittel, DominiqueWatier, BrunoKnoll, AloisWatkins, A. NealKnopp, DietmarWatson, Dennis G.Ko, HyounghoWatson, RobertKo, HyunhyubWatts, Michael J.Ko, Ren-songWebb, R. ChadKo, Tae JoWebb, ThomasKobayashi, MakikoWeber, AndreasKobsar, DylanWeber, RobertKocejko, TomaszWeber, William JosephKoch, ChristianWebster, JohnKocur, DusanWeddell, AlexKodera, NoriyukiWegener, JoachimKofman, JonathanWehrmeister, MarcoKoh, AhyeonWei, BoKoh, JaehanWei, Da-HuaKoh, Kah HowWei, GangKöhler, BerndWei, GuohuaKohut, PiotrWei, HemingKøien, Geir M.Wei, KentKoike, YasuharuWei, LeiKoizumi, AkioWei, MinKojima, FumihideWei, Pei-KuenKok, ManonWei, TaoKokkinos, PanagiotisWei, WeiKołakowska, AgataWeinberg, GrahamKolakowski, JerzyWeinberg, MarcKolias, ConstantinosWeis, JaredKommers, PietWeitzen, Jay A.Komninos, NikosWellard, MarkKomodromos, MichaelWeller, MichaelKomogortsev, OlegWells, B. EarlKoncar, VladanWells, IanKonečný, JaromírWelp, GerhardKong, Kien VoonWeltzien, CorneliaKong, Ling BingWen, ChengluKong, QingzhaoWen, Chih-YuKong, Siu-KaiWen, Chih-YungKong, Yong LinWen, GuanghuiKongsuphol, PattharaWen, GuojunKonh, BardiaWen, KunhuaKonieczny, ŁukaszWen, ShengKonishi, KuniakiWendel, JanKonovaltsev, AndriyWeng, Ruby Chiu-hsingKonstantaki, MariaWerneck, Marcelo MartinsKonstantas, DimitriWerner, PhilippKonstantinova, JelizavetaWerthschützky, RolandKonstantopoulos, CharalamposWessel, NielsKoo, DongyoungWesterdahl, DaneKoo, Hyung-JunWesterlund, TomiKootstra, GertWestra, JohannaKopáčik, AlojzWetterlind, JohannaKopel, PavelWetterö, JonasKopniak, PiotrWeyn, MaartenKoppal, SanjeevWhelan, Matthew J.Kopsaftopoulos, FotisWhite, CraigKopyt, PawełWhite, Richard M.Kor, Ah-LianWhite, Scott M.Korampally, VenumadhavWicks, MichaelKoren, KlausWieland, MatthiasKorhonen, IlkkaWierzba, PawełKorkali, MertWierzbicki, DamianKornuta, TomaszWierzbicki, SławomirKorobiichuk, IgorWietfeld, ChristianKorotchenkov, GenadyiWijnants, MaartenKorposh, SerhiyWilczyński, SławomirKorwan, DanielWild, GrahamKos, AntonWildmann, NormanKoskimäki, HeliWilhelm, ElisabethKosman, JoannaWilhelm, JayKosmopoulos, DimitriosWilhelm, StefanKošnar, KarelWilkens, MirjaKostamovaara, JuhaWilkins, RossKotiuga, MicheleWilkinson, BenKotropoulos, ConstantineWillersinn, DieterKotsifakos, AlexiosWilliams, BillyKottapalli, Ajay Giri PrakashWilliams, RonKottege, NavindaWilliams, TomKoubias, StavrosWillmott, JonKoucheryavy, AndreyWilm, JakobKoukoulas, SotiriosWilson, Alphus DanKoulamas, ChristosWilson, Andrew J.Koumoulis, DimitriosWilson, BenjaminKourkoumelis, NikolaosWilson, CyrilKourtiche, DjilaliWilson, Philip A.Kõuts, TarmoWilson, William L.Koutsomitropoulos, Dimitrios A.Wincza, KrzysztofKoutsoudis, AnestisWinkler, Thomas E.Koutsoukos, XenofonWipf, MathiasKoutsouris, DimitrisWippermann, FrankKouyoumdjieva, SylviaWiśniowski, PiotrKowalak, StanisławWitkowska Nery, EmiliaKowalczyk, KonradWitos, MiroslawKowalski, Piotr A.Wojaczek, PhilippKoyama, Christian N.Wojciechowski, AdamKoyama, ShoichiWojnowski, WojciechKozakov, RuslanWojtas, JacekKoziol, ScottWold, Jens PetterKraft, MarekWolf, WayneKraft, MartinWolfe, BrittonKrajnik, TomasWolff, MarcusKrajzewicz, DanielWöllenstein, JürgenKralevska, KatinaWon, Chee-SunKrankenhagen, R.Won, MyounggyuKrasnoslobodtsev, Alexey V.Wong, CharenceKrasteva, VesselaWong, Ka-LeungKratzke, NaneWong, Kok-SengKrause, ColleenWong, LawrenceKrause, ThomasWong, LeslieKravari, KalliopiWong, Ling TimKrawczuk, MarekWoo, HyenkyunKrawczyk, BartoszWoo, Jong-KwanKrejcar, OndrejWoo, Wai LokKretz, TobiasWood, BaydenKreuz, ThomasWoodfield, PeterKrief, FrancineWorch, RemigiuszKrishnan, LaxminarayananWotzka, DariaKrishnan, SridharWoungang, IsaacKristensen, AndersWoznowski, PrzemyslawKrižaj, JanezWu, Chao-chengKrolczyk, GrzegorzWu, ChenshengKrolczyk, JolantaWu, ChenshuKrólicka, AgnieszkaWu, Chung-minKronberger, RainerWu, Chung-Tse MichaelKrstulović-Opara, LovreWu, GuofenKrüger, BjörnWu, GuofengKrüger, FrankWu, GuoqiangKruger, Jan-LouisWu, JianfengKrull, Ulrich J.Wu, JiankangKruss, SebastianWu, JinKryjak, TomaszWu, Jin JeiKryszkiewicz, PawełWu, JingKrzeszowski, TomaszWu, LiliKrzysztof, NausWu, MingquanKrzywiecki, ŁukaszWu, QiKsiezopolski, BogdanWu, QiushengKtena, AphroditeWu, Shu-PaoKuang, YangWu, StephenKubba, Ali E.Wu, T.YKubíček, VojtěchWu, Wei-ChiangKübler, ThomasWu, Wen-JongKuckling, DirkWu, Wen-RongKudelski, AndrzejWu, XiaoshengKuhnhenn, JochenWu, XueruiKukke, Sahana N.WU, Yik-ChungKulathumani, VinodWu, YuanmingKulatunga, ChamilWu, ZhengKulesza, WlodekWu, ZhiluKulesza, ZbigniewWuest, ThorstenKulkarni, KedarWulamu, WasitKulma, MagdalenaWunderle, StefanKulmer, JosefWunderlich, ThomasKulyukin, VladimirWysocki, MarianKulzer, FlorianXenakis, ApostolisKumamoto, KazuoXenofon, StrakosasKumar, ManishXi, PengKumar, PardeepXi, XinKumar, Suryadevara NagenderXi, XugangKumar, UttamXia, ChunleiKumar, VimalXia, JunKumeria, TusharXia, WenfengKummer, WolfgangXia, YoushenKunihisa, TashiroXian, XiaojunKunkel, JulianXiang, DeliangKunze, KaiXiang, Limin Kuo, Cheng ChienXiang, XianboKuo, Chien-NanXiao, BinKuo, Chung-HsienXiao, DeqinKuo, ChungyenXiao, JiangKuo, Tsung-RongXiao, JianpingKuo, WatsonXiao, PerryKuo, Yaw-WenXiao, WenKuok, Sin-ChiXiao, XiongKupkova, LucieXiao, YuKurbatov, A.M.Xiao, ZhuKuremoto, TakashiXie, JinghangKurihara, ToruXie, LeiKurillo, GregorijXie, PuKurkina, TetianaXie, RanKurlyandskaya, Galina V.Xie, XinKuroda, RihitoXie, YingyingKuroki, ShinichiroXie, YongKurt, MehmetXie, ZhijianKurz, FranzXimenes, EduardoKurz, GerhardXing, FeiKurzatkowska, KatarzynaXing, XiaogangKusche, JürgenXiong, JipingKushki, AzadehXiong, XingguoKussul, NataliiaXu, BenKuznetsov, NikitaXu, ChungangKwak, Moon K.Xu, ChunyeKwak, YoungjooXu, Dong-ShengKwan, ChimanXu, FengKwartowitz, DavidXu, GuobaoKwiecień, JoannaXu, HaitaoKwon, Goo-RakXu, JianKwon, JunseokXu, JiangtaoKwon, Soon-HongXu, JiaqiangKyamakya, KyandoghereXu, JiawenKyriazis, NikolaosXu, LinaLa Forgia, NicolasXu, QingsongLa Manna, MarcoXu, ShengliLa, HungXu, Sheng-yongLabati, Ruggero DonidaXu, TianhuaLaBelle, JeffreyXu, WeiLabib, MahmoudXu, WeitaoLabiod, HoudaXu, WenLabra Gayo, José EmilioXu, WenyaoLabram, John G.Xu, XiangguoLad, Robert. JXu, XiaochuanLadha, CassimXu, XiaolinLaefer, DebraXu, XingLagkas, ThomasXu, YongLago, Claudimir Lucio DoXu, YuquanŁagód, GrzegorzXuan, HaifengLagrange, MathieuXuan, XiangchunLai, AntoniaXue, JinghaoLai, ChinLunXue, XinyuLai, Po-HsiangYaacoubi, SlahLai, StefanoYagasaki, KazuyukiLai, Wen ChengYaghoubi, HoumanLai, Ying-ChihYahiaoui, Riad.Lai, Yu-RenYamamoto, KyosukeLakshminarayana, PolavarapuYamamoto, ToshiyukiLal, ChhaganYamaner, Feysel YalçınLAM, Kwok-hoYamato, YojiLamarche, GeoffroyYamauchi, TakashiLamberti, FabrizioYan, (Ryan) Wai-ManLambrecht, StefanYan, HaomingLamonaca, F.Yan, MingyuanLamparelli, Rubens Augusto CamargoYan, Wai YeungLan, Kun-ChanYan, XinggangLan, TianYan, YuLanceros-Méndez, SenentxuYan, ZhepingLancis, Basam MuslehYáñez, Itzamá LópezLancry, MatthieuYáñez-Sedeño, PalomaLandaluce, HugoYang, BanghuaLandes, TaniaYang, BoLandini, ManueleYang, ChengLandowska, AgnieszkaYang, Chih-TsungLaneve, GiovanniYang, ChouchangLang, Hans PeterYang, Chuan-KaiLang, JochenYang, ChungangLang, WalterYang, DingtianLanga, SergiuYang, DongminLangley, PhilipYang, Eileen Chih-YingLangone, RoccoYang, GengLanorte, AntonioYang, GuilinLanza, JorgeYang, GuominLanza-Gutierrez, Jose M.Yang, HengzhaoLanzola, GiordanoYang, InseokLaory, IrwandaYang, JiachenLappi, OttoYang, JianjunLapray, Pierre-JeanYang, JieLaracca, MarcoYang, JinfengLarijani, HadiYang, KaiyuanLarsen, Jakob JuulYang, LiangliangLarsen, MortenYang, MaoLarsen, Peter GormYang, Mau-TsuenLas-Heras, FernandoYang, MingLashkari, BahmanYang, Ming-DerLaski, Pawel AndrzejYang, MingruiLasky, TyYang, ShifeiLasri, TuamiYang, ShikuanLatif, YasirYang, ShimingLattanzi, EmanueleYang, ShuhuiLattanzio, Veronica Maria TeresaYang, TaoLau, LawrenceYang, TingLau, MichaelYang, WeiLaudani, AntoninoYang, Wei-hsiungLauer, MartinYang, WentaoLaufer, ShlomiYang, XiLaurent, MarylineYang, XiaoyunLauridsen, MadsYang, XinmaiLaviada, JaimeYang, YangLaviale, MartinYang, YezhouLavine, BarryYang, YingchenLavric, AlexandruYang, YinyeLavrova, OlgaYANG, YixinLaw, Yee WeiYang, Yuan-SenLawson, BrianYang, YunzeLazar, DusanYang, Zhi-BoLazarescu, MihaiYanoviak, Stephen P.Lazaridis, PavlosYanushkevich, SvetlanaLazaro, AntonioYao, JasonLázaro, José L.Yao, JunLazarov, AndonYao, ShanshanLazarova-Molnar, SanjaYao, SuyingLazerges, MathieuYao, XiaLe Bars, FabriceYao, XuanxiaLe Bas, Pierre-Yves Yao, YaiboLe Borgne, HervéYao, YaoLe Carrou, Jean-LoïcYao, YimingLe Gac, SeverineYao, YuanLe Moullec, YannickYao, ZhengLe Page, MichelYapici, Murat KayaLe, Duc VanYared, RamiLe, Nam TuanYashchyshyn, YevhenLe, Trung-KienYasmin, ShamsunnaharLe, TuanYatabe, RuiLe, Tuan AnhYe, WeilinLe, Viet DucYE, XujunLeach, RichardYe, YuLebastard, VincentYe, YuanLebel, KarinaYee, Shannon K.LeBlanc, GabrielYeh, Kuo-HuiLeBlanc, Heath J.Yeh, Sheng-ChengLeccese, FabioYeh, Yi-ChunLecomte, SteveYehia, SherifLecours, VincentYelamarthi, KumarLecoutere, JeroenYeoh, PheeLee, AhyoungYeom, EunseopLee, Boon GiinYeom, SeokwonLee, Byung ChulYeow, Chen HuaLee, ChanggilYi, ChuLee, ChangwonYi, JiaziLee, Chao-HsienYildirim, Kasim SinanLee, Cheng-ChiYildiz, Huseyin UgurLee, Cheng-MingYilmaz, HuzeyfeLee, Dae-JinYilmaz, MehmetLee, Dae-SikYim, SehyukLee, DonghwaYim, Un HyukLee, DonghyunYin, GuodongLee, Dong-YeonYin, GuoLuLee, Gil SikYin, JifuLee, Gil-YongYin, JunLee, Gyu MyoungYin, PenghangLee, Hee YoungYin, ShouyiLee, Hee-JoYin, WuliangLee, HyokiYoerger, DanaLee, HyungJuneYohanes, KhosiawanLee, HyunjoonYokota, TomoyukiLee, Il-GuYong, Yang-chunLee, InbokYoo, ByungseokLee, Jae OneYoo, Han WoongLee, Jang-MyungYoo, JunsooLee, JejungYoon, ChangwooLee, JisunYoon, GilwonLee, Jong-HeunYoon, Hyeun JoongLee, JuheonYoon, HyungchulLee, JungchulYoon, Ji-WookLee, Jung-RyunYoon, SangpilLee, JunyoungYoon, SeokhoonLee, Kang-YoonYoon, Yeo-SunLee, KeekeunYoon, YongsoonLee, KeunhwaYork, TimothyLee, KevinYoshimura, TakeshiLee, Ki YongYoshioka, HiroakiLee, MarkYoshizawa, ShingoLee, Milton L.You, Jung-MinLee, Moon GuYoun, Duck HyunLee, Sang-gonYousaf, AdnanLee, SangyounYousefi, SiavashLee, Seok JaeYoussofzadeh, VahabLee, Seung-BeckYperzeele, LaetitiaLee, Suk JinYu, Chin-pingLee, Sung PilYu, ChunyangLee, Sunghoon IvanYu, DantongLee, SungyoungYu, Feiqiao BrianLee, TaikjinYu, GuandingLee, Tian-FuYu, GuizhenLee, Tsung-HsuehYu, Gwo-JongLee, Wee SitYu, HaoyongLee, Wei-NingYu, HuapengLee, Woo-KyungYu, JiayongLee, WooyoungYu, Jong-WonLee, Yang-hanYu, KangLee, YangsunYu, RongLee, YonghoYu, Ting-ToLee, Yong-WookYu, WeirenLee, Young JooYu, XingeLee, YoungDooYu, XunLee, Young-heeYu, YanguangLees, John E.Yu, YunLefeuvre, ElieYu, ZhiwenLefèvre, EricYuan, BingLeger, Jean-MichelYuan, ChengzhiLegrand, AlexandreYuan, DajingLei, YaguoYuan, DongLei, YangYuan, LeiLei, ZhenkunYuan, QiangqiangLeightley, DanielYuan, QilongLein, JamesYUAN, RENMAOLeister, WolfgangYuan, XiaohuiLeitao, Diana C.Yuce, Mehmet R.Lekkas, Anastasios M.Yuen, ChauLellmann, JanYuffa, Alex J.Lemic, FilipYun, MiraLenci, StefanoYun, UnilLengden, MichaelYun, YoungsunLenzo, BasilioYunusa-Kaltungo, AkiluLeonardi, FrancescaZabulis, XenophonLeón-Luis, Sergio F.Zafra, AmeliaLepidi, MarcoZagar, BernhardLepora, NathanZagorovsky, KyrylLeporati, FrancescoZaitsev, Dmitry L.Lepore, MariaZak, AllaLepot, MathieuZalejska-Jonsson, AgnieszkaLerchner, JohannesZalyubovskiy, VyacheslavLeroux, DenisZamora, GerardLeroux, PaulZamora-Martinez, FranciscoLesch, AndreasZampetti, EmilianoLesiak, PiotrZang, YapingLeukhin, AnatoliiZannin, EmanuelaLeutenegger, MarcelZanotti Fragonara, LucaLevanon, NadavZanuttigh, PietroLeveneur, JeromeZapién, Hiram GaleanaLévesque, LucZappatore, MarcoLevi, AlessandroZarejousheghani, MashaalahLewis, RogerZarifi, Mohammad H.Lhotáková, ZuzanaZarifi, Mohammad HosseinLi, AihuaZarkoganni, KonstantiaLi, BaihuaZarpelão, Bruno BogazLi, BaoqiangZarra, TizianoLi, BeiwenZatar, WaelLi, BoZatelli, PaoloLi, BofengZawadzki, JarosławLi, ChangzhiZdunek, ArturLi, ChongZeadally, SheraliLi, ChuanjiangZehentner, NorbertLi, DachuanZembaty, ZbigniewLi, DaoliangZeng, HulieLi, DapengZeng, MingLi, DerenZeng, QinghuaLi, DeshiZeng, ShuwenLi, FanZeni, LuigiLi, FangZennaro, MarcoLi, FangminZeydan, EnginLi, GangZhan, NaiqianLi, GenZhan, XiwuLi, HaoZhang, BaochengLi, Heng-ChaoZhang, ChunweiLi, HongkunZhang, CongchunLi, JiaZhang, DanLi, JianZhang, DeganLi, JianzhaoZhang, DeyuanLi, JinZhang, DimingLi, JingZhang, DongzhiLi, JunhaoZhang, DunLi, KaiweiZhang, EnxiaLi, LiangZhang, FanLi, MenghuiZhang, FeihuLi, MiaoZhang, FengjiaoLI, MinZhang, GuangyuLi, Ming-HuangZhang, GuoLi, MoZhang, GuoshengLi, NingZhang, GuozhiLi, Paul C.H.Zhang, HaijunLi, PeterZhang, HanLi, QiaoZhang, HankuiLi, QiliangZhang, HeyeLi, QingguoZhang, HongmeiLi, RuyaZhang, HuamingLi, SinanZhang, HuiLi, TianchengZhang, HuihuiLi, WeiZhang, JiashuLi, WeijieZhang, JieLi, WeiyangZhang, JingjingLi, WenfengZhang, JingtuoLi, WenjuanZhang, JingxiongLi, WenliangZhang, JingzhouLi, WenlingZhang, JitaoLi, XiaoZhang, JixianLi, Xiaohua (Edward)Zhang, JunshengLi, XiaopengZhang, KefeiLi, XinZhang, LeLi, XingxingZhang, LefeiLi, XuejunZhang, LeiLi, XueqingZhang, LiangpeiLi, YangZhang, LibingLi, YingguangZhang, LifuLi, YundongZhang, LinLi, Yung-HuiZhang, LiqiangLi, ZhanZhang, MeiningLi, ZhaoliangZhang, MinLi, ZheZhang, PengyuLi, ZhenhongZhang, QianniLi, ZhenyuZhang, QijinLi, ZhixiongZhang, QingLI, ZhongliangZhang, RuiLiang, Chiu-kuoZhang, ShaolinLiang, HuiZhang, ShuaiLiang, JingZhang, ShushengLiang, JinxingZHANG, SongsongLiang, Jun-RuiZhang, SuLiang, LuZhang, TingtingLiang, MengbingZhang, WeiLiang, RongguangZhang, Wenjun (Chris)Liang, StevenZhang, WenlongLiang, Tsair-ChunZhang, WenshengLiang, XishuangZhang, WentaoLiang, XuefengZhang, WenyuLiao, BinZhang, XianfengLiao, ChangruiZhang, XiankuLiao, Jyh-feiZhang, XianmingLiao, Kuo-ChihZhang, XiaotongLiao, Lun-DeZhang, XinLiao, Shu-HsienZhang, XinMingLiao, Tai-ShanZhang, XudongLidgi-Guigui, NathalieZhang, XueboLie, ArneZhang, XuetaoLieberzeit, PeterZhang, XunjunLiebig, ThomasZhang, Ya-nanLiebisch, FrankZhang, YanjieLieser, JanZhang, YanningLiguori, AnnaZhang, YanpingLikamwa, Patrick L.Zhang, YanxiaLilienthal, HolgerZhang, YifengLillethun, DaveZhang, YileiLim, Dong-WonZhang, Yimin DanielLim, GeunbaeZhang, YinLim, Hyung JinZhang, YongLim, Jae-HanZhang, YuLim, Kian-MengZhang, YudongLim, KihoZhang, YumingLim, SejoonZhang, YunLim, Si-HyungZhang, ZhenbinLim, SungjoonZhang, ZhenjiangLim, Yah LengZhang, ZimingLim, Yee YanZhao, CezhouLima, João PauloZhao, DiLima, JoséZhao, DongfangLima, RuiZhao, DongyaLin, Bor-ShingZhao, JingzhouLin, Bor-ShyhZhao, JuziLin, Chang HongZhao, KaichunLin, ChiZhao, LiangLin, Chih-mingZhao, LiboLin, Chih-pingZhao, LifanLin, Chii-WannZhao, MingjunLin, Chin-FengZhao, PengLin, Ching-JuZhao, PingLin, Chi-YingZhao, ShengkuiLin, Chu-EnZhao, SihaoLin, Chun-ChengZhao, WenbingLin, Chun-LingZhao, WenfengLin, DeyanZhao, WenkeLin, FengZhao, XiangyongLin, GungunZhao, XihongLin, Hung-YinZhao, YangLin, Jing (Janet)Zhao, YanjunLin, JonathanZhao, YiLin, Jr-LungZhao, YubinLin, Ming-BoZhao, ZeyuLin, QianqianZhao, ZhanLin, Shih-ChunZheng, BangyouLin, TachunZheng, ChengshiLin, Tsung-HungZheng, EnliangLin, Tzu-KangZheng, KanLin, WeiZheng, LiangLin, XiangguoZheng, ShijieLin, Yang-weiZheng, XianweiLin, Yaw-jenZheng, Xin TingLin, Yuan-PinZheng, YanLin, Yuh-ChungZheng, YuanjinLin, ZhibinZhong, LihengLin, Zong-HongZhong, Yu-WuLind, Pedro G.Zhou, BingLinden, MariaZhou, ChiLindenbergh, RoderikZhou, HaiboLindino, Cleber AntonioZhou, JiafengLindner, GerhardZhou, JianfengLindquist, NathanZhou, JianjiangLing, Keck VoonZhou, LiangLing, SteveZhou, MengChuLing, Wing-KuenZhou, Ming-tuoLingua, Andrea MariaZhou, QifanLinn, Jimmy B.Zhou, QinLinnamo, VesaZhou, QingxiangLins, AndréZhou, QuLinß, SebastianZhou, ShengLinty, NicolaZhou, ShikunLinzon, YoavZhou, WeiLiotta, AntonioZhou, XiaLiou, Han-TsungZhou, XiangyangLiou, MichelleZhou, Xiao DongLippiello, VincenzoZhou, XinLisak, GrzegorzZhou, YongchengLisauskas, AlvydasZhou, YongjinLissenden, Clifford J.Zhou, YufengLitti, LucioZhou, ZhenyuLiu, BingbingZhu, BowenLiu, BoZhu, ChunshengLiu, Chi (Harold)Zhu, FengLiu, Chien-ShengZhu, George Z.H.Liu, ChuanjunZhu, HaoshenLiu, ChunweiZhu, JiguiLiu, DapengZhu, Ka-diLiu, DatongZhu, LikaiLiu, GuigenZhu, LingliLiu, HongZhu, LiqiangLiu, HongboZhu, RuiLiu, HonghaiZhu, ShanLIU, HongweiZhu, ShaoliLiu, Hung-HuanZhu, XiangdongLiu, JingZhu, XiaoshanLiu, JingquanZhu, YungangLiu, JinyunZhu, YuqingLiu, JuewenZhu, ZheLiu, JunZhu, ZhigangLiu, KunZhu, ZhijieLiu, LiangZhuang, HanqiLiu, LianxiZhuang, JunLiu, MeiqinZhuang, YanyanLIU, MenglongZhuang, YuanLiu, MingZhuge, XiaodongLiu, Pang-WeiZi, YunlongLiu, PeilinZiabari, AmirkoushyarLiu, PeixiangZiarek, Lukasz (Luke)Liu, QingjunZidansek, AleksanderLiu, QingkunZijlstra, PeterLiu, QiongZikria, Yousaf BinLiu, QuanhuaZilic, ZeljkoLiu, RanZimmer, LucLiu, ShaopengZimmermann, StefanLiu, Shing-HongZin, Thi ThiLiu, Shi-XiaZingaretti, PrimoLiu, SicongZivcak, MarekLiu, SijiaZiviani, ArturLiu, TaoŽivković, Emila M.Liu, Tzong-shiZografopoulos, DimitriosLiu, WanliZografopoulos, Dimitrios C.Liu, Wei-ChengZolghadri, AliLiu, Wei-MinZoli, MarcoLiu, WeitingZolotoukhina, TatianaLiu, WeiweiZoltan, KantorLiu, WeiXianZorbas, DimitriosLiu, WenZotovic Stanisic, RankoLiu, Wen-FungZou, Fangxin (Frank)Liu, WenzhongZou, HanLiu, XintaoZou, SaiLiu, XuanZou, XiaoboLiu, Ya-FengZou, XudongLiu, YangZou, YongjinLiu, YaohuaZubia, JosebaLiu, YongZucali, MaddalenaLiu, YonggangZuccheretti, EnricoLiu, YongkangZucco, MassimoLiu, YongliangZuffada, CinziaLiu, YuKangZug, SebastianLiu, Yung-tienZuo, BoxinLiu, ZenghuaZuo, LihuaLiu, ZhaoZuppella, PaolaLiu, ZhaorongZus, FlorianLiu, ZhidanZych, MarcinLiu, Zhigang


